# Protein solubility and differential proteomic profiling of recombinant *Escherichia coli *overexpressing double-tagged fusion proteins

**DOI:** 10.1186/1475-2859-9-63

**Published:** 2010-08-28

**Authors:** Chung-Hsien Cheng, Wen-Chien Lee

**Affiliations:** 1 Department of Chemical Engineering, Systems Biology and Tissue Engineering Research Center, National Chung Cheng University, Chiayi, 621, Taiwan

## Abstract

**Background:**

Overexpression of recombinant proteins usually triggers the induction of heat shock proteins that regulate aggregation and solubility of the overexpressed protein. The two-dimensional gel electrophoresis (2-DE)-mass spectrometry approach was used to profile the proteome of *Escherichia coli *overexpressing N-acetyl-D-glucosamine 2-epimerase (GlcNAc 2-epimerase) and *N*-acetyl-D-neuraminic acid aldolase (Neu5Ac aldolase), both fused to glutathione S-transferase (GST) and polyionic peptide (5D or 5R).

**Results:**

Overexpression of fusion proteins by IPTG induction caused significant differential expression of numerous cellular proteins; most of these proteins were down-regulated, including enzymes connected to the pentose phosphate pathway and the enzyme LuxS that could lead to an inhibition of tRNA synthesis. Interestingly, when plasmid-harboring cells were cultured in LB medium, gluconeogenesis occurred mainly through MaeB, while in the host strain, gluconeogenesis occurred by a different pathway (by Mdh and PckA). Significant up-regulation of the chaperones ClpB, HslU and GroEL and high-level expression of two protective small heat shock proteins (IbpA and IbpB) were found in cells overexpressing GST-GlcNAc 2-epimerase-5D but not in GST-Neu5Ac aldolase-5R-expressing *E. coli*. Although most of the recombinant protein was present in insoluble aggregates, the soluble fraction of GST-GlcNAc 2-epimerase-5D was higher than that of GST-Neu5Ac aldolase-5R. Also, in cells overexpressing recombinant GST-GlcNAc 2-epimerase-5D, the expression of σ^32 ^was maintained at a higher level following induction.

**Conclusions:**

Differential expression of metabolically functional proteins, especially those in the gluconeogenesis pathway, was found between host and recombinant cells. Also, the expression patterns of chaperones/heat shock proteins differed among the plasmid-harboring bacteria in response to overproduction of recombinant proteins. In conclusion, the solubility of overexpressed recombinant proteins could be enhanced by maintaining the expression of σ^32^, a bacterial heat shock transcription factor, at higher levels during overproduction.

## Background

Under the regulation of strong promoters, as in numerous commercial plasmid-based vectors, heterologous proteins are typically expressed at high levels in *Escherichia coli*. The overexpression of plasmid-encoded genes can trigger transcription of heat-shock genes and other stress responses and often result in the aggregation of the encoded proteins as inclusion bodies [1]. The formation of inclusion bodies offers distinct advantages for the separation of overexpressed protein, because the aggregates that mostly contain the product in a high concentration can be easily isolated [2]. However, the recombinant proteins found in inclusion bodies are often in a misfolded state, methods that can be used to avoid aggregation to yield a soluble and active product are sometime very desirable. To improve the expression of soluble recombinant proteins, introducing a fusion partner (tag) such as N-utilization substance A (NusA), maltose-binding protein (MBP), thioredoxin (TRX), or glutathione S-transferase (GST), to the recombinant protein is one of the most commonly used methods to increase solubility [3,4]. We previously constructed two double-tagged gene fusions for overexpressing N-acetyl-D-glucosamine 2-epimerase (GlcNAc 2-epimerase) and *N*-acetyl-D-neuraminic acid aldolase (Neu5Ac aldolase), two sequential enzymes in the production of sialic acids. Both proteins were tagged with GST at the N-terminus, but at the C-terminus, one was tagged with five contiguous aspartate residues (5D) and the other with five contiguous arginine residues (5R) [5]. The fusions were so designed to yield fusion proteins having charged surfaces at working pH, which allowed isolation and immobilization in a single step with either an anionic or a cationic exchanger that electrostatically bound fusion proteins via the 5D or 5R tag. In contrast to overexpressed GST alone that was totally soluble, however, most of overexpressed fusion proteins were in insoluble fraction. Although these fusion proteins overexpressed in *E. coli *were enzymatically active in both soluble and insoluble (aggregate) fractions. The present paper thus delineates the proteomic profiles of overproducing bacteria and presents results that could be useful for conceive a strategy to improve the production of soluble recombinant proteins.

Recombinant protein overexpression has been known to induce significant physiological changes such as the stress response to heat-shock in *E. coli *[6]. The presence of the inducer isopropyl-β-D-1-thiogalactopyranoside (IPTG) alone can even influence *E. coli *metabolism substantially, altering the synthesis of certain proteins [7]. When a recombinant protein is expressed at high rates, the system of cytosolic chaperones and proteases in bacteria is presumably induced to express in an altered pattern, in comparison with the host cells without overexpressing recombinant proteins. In addition to facilitating the folding of nascent proteins, several molecular chaperones and heat shock proteins are induced to inhibit the formation of inclusion bodies by reducing aggregation and promoting proteolysis of misfolded proteins. The simultaneous overexpression of chaperone/heat shock protein encoding genes and recombinant target proteins proved effective in several instances [8]. To increase the solubility of recombinant proteins, the co-overproduction of individual chaperones as well as the combined overproduction of the functionally cooperating chaperone network of the *E. coli *cytosol has been attempted [9]. Based on experimental results, Garcia-Fruitos et al. suggested that the so-called *E. coli *quality control system (made up of chaperones and proteases) acts coordinately to promote solubility at the expense of conformational quality [10]. A study of global changes in protein expression that occur in response to the rapid synthesis of a recombinant protein would therefore help to elucidate the mechanisms regulating recombinant protein solubility.

Proteomic analysis has been employed to compare changes in the expression levels of cellular proteins under particular genetic and environmental conditions. The conventional approach to proteomics is a combination of high-resolution two-dimensional gel electrophoresis (2-DE) to separate the proteins and mass spectrometry (MS) to identify each isolated protein. Proteomic studies have helped elucidate complex cellular responses such as starvation, temperature shock, and stress responses in *E. coli *and they have facilitated its use in a variety of biotechnological applications [11]. Knowledge of basic cellular processes provides the basis for developing methods to better control heterologous protein expression [12]. Proteomic analysis has been used to disclose cellular protein changes during the overexpression of heterologous proteins in *E. coli *under different fermentation conditions [13-16]. Strategies to increase the production of serine-rich proteins and enhance cytosolic or secretory protein production have been proposed based on the disclosed proteome profiles [17,18].

## Results and Discussion

### Differential expression profiling of *E. coli *induced with IPTG for protein overexpression

**Figure 1:**
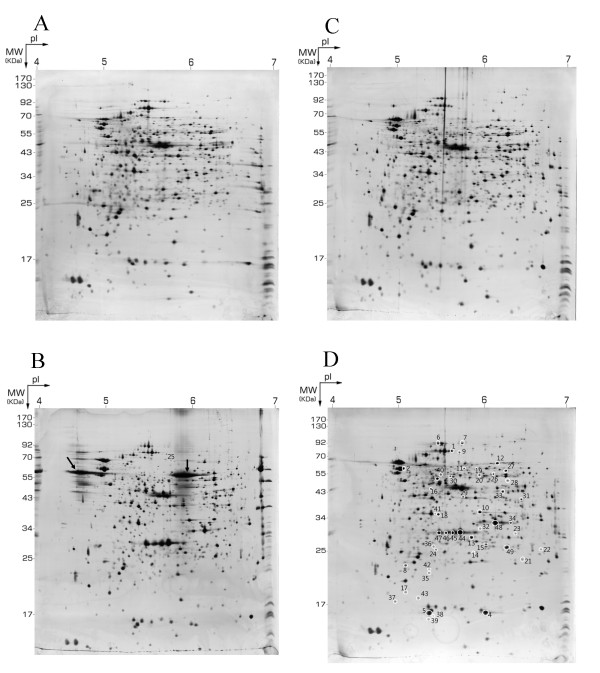
**Representative 2-DE gels of *E. coli *BL21 plasmid-bearing strains**. Two-dimensional gel electrophoresis (2-DE) of the cell lysates of *E. coli *BL21 harboring pGEX-2TK-nanA-5R (A, B) and *E. coli *BL21 harboring pGEX-2TK-2ep-5D (C, D) harvested before (A, C) and after 3-h IPTG induction (B, D). Numbers on the gels reflect the identified protein spots as denoted in Table 1.

The bacteria used in this study are the host *E. coli *BL21 and *E. coli *BL21 strain harboring pGEX-2TK, pGEX-2TK-nanA-5R and pGEX-2TK-2ep-5D, which encoded GST, GST-Neu5Ac-aldolase-(arginine)_5 _and GST-GlcNAc 2-epimerase-(aspartate)_5_, respectively [5]. These four bacteria that were cultivated in Luria-Bertani (LB) medium showed a similar growth pattern, even throughout the expression of recombinant proteins induced by the addition of IPTG (Additional file 1). When the OD_600 _reached 0.8 (denoted as time zero or T0), a sample was taken for proteome analysis and IPTG was added to both host-strain and plasmid-bearing cell cultures. After a 3-h induction, the recombinant protein was overexpressed to a substantial level and at that time another sample (denoted T3) was taken and compared with sample T0 of the same bacteria. To optimize the resolution of the proteome, pH 4-7 IPG strips were employed to cover the range in which most proteins of *E. coli *focus isoelectrically. Overexpressed GST and double-tagged Neu5Ac-aldolase were seen in the gels, but double-tagged GlcNAc 2-epimerase was not detected in the gels because it was not absorbed into the IEF strip for some unknown reasons. There were totally about 900 protein spots detected on 2-DE gels by MS-compatible silver staining. Three replicate runs were performed on the proteome of each *E. coli*. Figure 1 shows representative 2-DE images of the cellular proteome of *E. coli *cells harboring pGEX-2TK-nanA-5R and pGEX-2TK-2ep-5D before and after IPTG induction for 3 h. The representative 2-DE images for the host (*E. coli *BL21) and *E. coli *BL21 harboring pGEX-2TK before and after IPTG induction are shown in Additional file 2. The total number of differentially expressed spots appearing in at least one of the four bacteria was 293 based on a significance level of *p *< 0.05; the number of differentially expressed spots became 136 when the significance level was set at *p *< 0.01. Two small heat shock proteins, IbpA and IbpB, and six AmpC spots were excluded from these counts. Among these spots, 49 that were differentially expressed (P < 0.05 and a fold-change around two or greater) corresponding to 44 proteins, as shown in Table 1. Also, time courses of the expression levels of these differentially expressed proteins in the host strain and in *E. coli *BL21 harboring pGEX-2TK-2ep-5D are shown in Additional file 3.

**Table 1:** List of identified proteins with altered expression after induction.

**Spot No**.	Gene name	**NCBI or SwissProt accession no**.	Description of protein	**Classification**^**a**^	Theoretical		**Fold change**^**b**^			
						
					M.W. (Da)	pI	BL3/BL0	2TK3/2TK0	NA3/NA0	EP3/EP0
1	*clpB*	gi|15832709	Protein disaggregation chaperone	H	95697	5.37	1.01	1.32	1.63	2.19*
2	*groEL*	gi|15804735	Chaperonin GroEL	H	57447	4.81	1.24	1.70*	1.23	1.73*
3	*hslU*	gi|170769640	Heat shock protein HslVU, ATPase subunit HslU	H	49678	5.24	0.96	2.20	0.70	3.5*
4	*ibpA*	gi|15804286	Heat shock protein IbpA	H	15764	5.77	n.d.^c^	∞^d^	∞	∞
5	*ibpB*	gi|30064996	Heat shock protein IbpB	H	16083	5.19	n.d.	∞	∞	∞
6	*acnB*	gi|170683451	Aconitate hydratase 2	C	91078	5.22	1.56	0.70	1.21	1.5*
7	*aceE*	gi|15799798	Pyruvate dehydrogenase subunit E1	C	99948	5.46	0.19*	0.67	0.13*	0.59
8	*atpH*	gi|145398	ATPase delta-subunit	C	19420	4.93	0.61	0.65	0.63*	0.56*
9	*maeB*	gi|222157177	NADP-dependent malic enzyme	C	82886	5.34	1.48	3.18*	2.82	2.20*
10	*mdh*	gi|440049	Malate dehydrogenase	C	29527	5.73	2.08*	1.37	1.39*	1.11
11	*pckA*	gi|16131280	Phosphoenolpyruvate carboxykinase	C	59891	5.46	1.28	1.14	1.17	1.34*
12	*sdhA*	gi|193065522	Succinate dehydrogenase, flavoprotein subunit	C	65024	5.85	1.02	2.89*	2.15*	1.90
13	*adk*	gi|15800203	Adenylate kinase	N	23628	5.55	0.84	0.77	0.69*	0.77*
14	*apt*	gi|16128453	Adenine phosphoribosyltransferase	N	19847	5.26	0.66*	0.53*	0.59*	0.62*
15	*dcd*	gi|15802547	Deoxycytidine triphosphate deaminase	N	21352	5.62	1.15	0.45	6.00*	7.83*
16	*deoB*	gi|91214099	Phosphopentomutase	N	44707	5.15	0.36	0.25*	0.25*	0.25*
17	*dut*	gi|16131511	Deoxyuridinetriphosphatase	N	16202	5.03	0.87	0.63*	0.42*	0.38*
18	*prsA*	gi|15801436	Ribose-phosphate pyrophosphokinase	N	34401	5.23	0.61*	0.49*	0.71*	0.54*
19	*purH*	gi|26250778	Bifunctional phosphoribosylaminoimidazolecarboxamide formyltransferase/IMP cyclohydrolase	N	57656	5.66	1.00	0.86	2.47*	1.43
20	*melA*	gi|15804711	Alpha-galactosidase	CH	51219	5.58	7.58*	1.80*	2.21*	1.74*
21	*gmhA*	gi|26106668	Phosphoheptose isomerase	CH	27392	8.24	0.96	2.09*	0.32*	0.43*
22	*lacA*	gi|15800071	Galactoside O-acetyltransferase	CH	22965	6.38	20.37	∞	∞	∞
23	*rbsB*	gi|15804351	D-ribose transporter subunit RbsB	CH	30919	6.85	3.20*	3.01	2.69*	0.70
24	*rpiA*	gi|15803449	Ribose-5-phosphate isomerase A	P	22903	5.20	1.61*	0.54*	0.64*	0.54*
25	*tktA*	gi|193063507	Transketolase	P	72464	5.43	2.88	n.d.	0.30*	n.d.
26	*dppA*	gi|193068548	Dipeptide ABC transporter, periplasmic dipeptide-binding protein	X	60467	6.21	2.75*	1.25	1.83	1.84*
27	*oppA*	gi|15801469	Oligopeptide transport; periplasmic binding protein	X	61099	5.95	2.58*	2.41*	1.96*	1.40*
28	*tnaA*	gi|15804305	Tryptophanase	X	53791	5.88	6.40*	2.65*	1.77*	1.39
29	*gcvT*	gi|16130807	Aminomethyltransferase, tetrahydrofolate-dependent, subunit (T protein) of glycine cleavage complex	X	40235	5.36	1.93*	1.05	0.90	1.40*
30	*glpK*	gi|15804515	Glycerol kinase	X	57226	5.36	0.24	0.34*	0.26*	0.77
31	*gatD*	gi|91211377	Galactitol-1-phosphate dehydrogenase	X	37874	5.94	1.85	1.46	1.98*	0.75
32	*garR*	gi|89109891	Tartronate semialdehyde reductase	X	30754	5.58	n.d.	n.d.	1.94*	n.d.
33	*ackA*	gi|1359437	Acetate kinase	X	43488	5.76	0.53	0.55*	0.64*	0.55*
34	*panC*	gi|16128126	Pantothenate synthetase	X	31692	5.91	1.57	0.73	1.87	3.13*
35	*luxS*	gi|15803206	S-ribosylhomocysteinase	X	19603	5.18	1.84	0.70*	0.53*	0.45*
36	*hdhA*	gi|15802033	7-alpha-hydroxysteroid dehydrogenase	X	26990	5.22	1.19	0.40*	0.61*	0.59*
37	*iscU*	gi|15803056	Scaffold protein	X	14011	4.82	0.70	0.68	0.36*	0.31*
38	*minE*	gi|15801396	Cell division topological specificity factor MinE	X	10286	5.15	2.00*	0.82	0.47*	0.88
39	*uspA*	gi|15804030	Universal stress protein	X	16113	5.11	3.60*	1.10	0.42*	0.78
40	*ilvC*	B1X9Z0	Ketol-acid reductoisomerase	X	54376	5.20	1.12	0.65	0.37*	0.74
41	*ybl119*	gi|242378351	Periplasmic binding protein and sugar binding domain, lacI family	X	33786	5.68	2.19	∞	∞	2.48
42	*yceI*	gi|218704465	Polyprenyl-pyrophosphate binding protein	X	20886	5.56	1.82	0.54*	0.40*	0.47*
43	*ydhR*	gi|170680041	YdhR protein	X	11297	5.09	1.10	0.57*	0.43*	0.63*
44	*ampC*	gi|41817	Beta lactamase TEM6	R	31646	5.93	n.d.	11.28*	5.41*	7.12*
45	*ampC*	gi|41817	Beta lactamase TEM6	R	31646	5.93	n.d.	∞	∞	∞
46	*ampC*	gi|41817	Beta lactamase TEM6	R	31646	5.93	n.d.	∞	∞	∞
47	*ampC*	gi|41817	Beta lactamase TEM6	R	31646	5.93	n.d.	∞	∞	∞
48	*ampC*	gi|41817	Beta lactamase TEM6	R	31646	5.93	n.d.	8.09*	42.78*	51.26*
49	*ampC*	gi|41817	Beta lactamase TEM6	R	31646	5.93	n.d.	3.83	7.05*	6.02*

^a^Classification: H, chaperone & heat shock protein; C, central metabolism and TCA cycle; N, nucleotide and nucleoside metabolic process; CH, carbohydrate metabolic and catabolic processes; P, pentose phosphate pathway; X, other function or pathway; R, antibiotic resistance.

^b^Relative %volume of 3-h sample/0-h sample in each group. BL, *E. coli *BL21; 2TK, *E. coli *BL21 harboring pGEX-2TK; NA, *E. coli *BL21 harboring pGEX-2TK-nanA-5R; EP, *E. coli *BL21 harboring pGEX-2TK-2ep-5D. The fold change was calculated by dividing the value of 3-h sample by the value of 0-h sample. If this number was less than one, the fold change is the reciprocal of this number (e.g. 0.70 is reported as 1.43 fold down-regulation).

^c^n.d., not detected.

^d^∞: present only in the 3-h sample.

* denotes significant differential expression.

Please note that the leaky expression of recombinant proteins, i.e., protein expression with addition of IPTG, was minimal in the plasmid-harboring cells. Upon IPTG induction, the number of up- and down-regulated spots was comparable in the host strain. In the plasmid-harboring cells, however, the differentially expressed spots were overwhelmingly down-regulated by IPTG induction, suggesting that the overexpression of recombinant proteins caused an unusually low expression of cellular proteins. As shown in Table 1, most down-regulated proteins in the plasmid-harboring cells were also found to be down-regulated in the host strain. Proteins up-regulated by IPTG were mainly the chaperone GroEL, galactoside O-acetyltransferase (LacA) and α-galactosidase (MelA). These results obtained with a proteomic approach are in good agreement with gene expression data of RNA levels of *E. coli *that was cultured in LB medium and treated with IPTG, which resulted in a high level induction of the *lacZYA *and *melAB *operons [19]. In addition, oligopeptide transport (OppA) and dipeptide ABC transporter (DppA) were up-regulated, suggesting an unusual requirement for nutrients by the bacterium during IPTG induction. Similarly, the strong up-regulation of the oligopeptide-binding protein OppA was found in a recombinant *Bacillus megaterium *strain overexpressing dextransucrase [20].

According to the study by Peng and Shimizu [21] protein abundance detected by 2-DE correlated well with enzyme activity in *E. coli *K12. Our proteomic analysis revealed that some enzymes like deoxyuridinetriphosphatase (Dut), 7-alpha-hydroxysteroid dehydrogenase (HdhA) and ketol-acid reductoisomerase (IlvC) showed reduced expression only in the plasmid-harboring cells, suggesting that cellular activities were blocked to some extent by the overproduction of plasmid-encoded proteins. The overproduction of recombinant proteins also caused a decrease in the level of a key enzyme (IlvC) involved in valine and isoleucine biosynthesis, similarly to results from a microarray study of overproduction of the α-subunit of luciferase in *E. coli *[22]. Furthermore, some proteins like the gluconeogenic enzyme NADP-dependent malic enzyme (MaeB) and the TCA cycle enzyme succinate dehydrogenase (SdhA) were significantly up-regulated after IPTG induction in plasmid-harboring cells. The up-regulation of the TCA cycle enzyme demonstrates the importance of the TCA cycle for the increased biosynthetic activity required by high-level protein synthesis [15]. Elevated levels of SdhA were also found in prior reports on the overproduction of another recombinant protein (leptin) [17].

**Figure 2:**
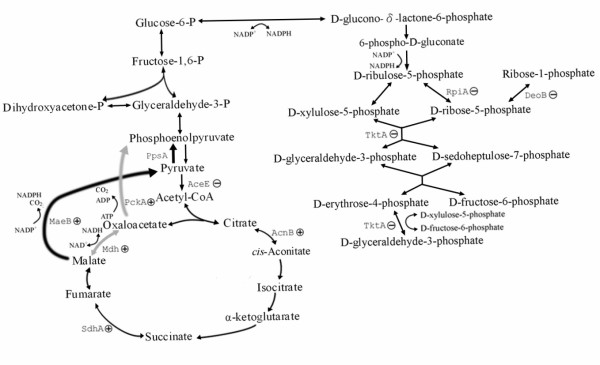
**The glycolytic pathway, the gluconeogenic pathway, the TCA cycle, and the pentose phosphate pathway in *E. coli *cultured in LB medium**. Up- and down-regulated proteins resulting from the proteome profiling are marked with ⊕ and ⊖, respectively.

For culturing *E. coli*, LB is a complex rich medium that likely contains glycolytic and gluconeogenic carbon sources [23]. As a gluconeogenic medium, amino acids and other small metabolites in LB fuel directly into the Krebs (TCA) cycle [24]. After IPTG induction, more gluconeogenesis takes place in order to generate glucose phosphate for the pentose phosphate pathway. The gluconeogenesis pathway however, was quite different in host and plasmid-harboring cells. Figure 2 was drawn based on the differentially expressed proteins relating to glycolysis/gluconeogenesis, the TCA cycle, and the pentose phosphate pathway (PPP). In the host strain, gluconeogenesis most likely occurred via malate dehydrogenase (Mdh) and phosphoenolpyruvate carboxykinase (PckA) (gray arrows), while in the plasmid-harboring cells, gluconeogenesis proceeded mainly through MaeB (boldfaced arrows). The enzyme MaeB catalyzes the formation of pyruvate and CO_2 _from malate in the generation of NADPH from NADP^+^. Pyruvate is further converted to phosphoenolpyruvate by the enzymatic action of phosphoenolpyruvate synthase (PpsA) in the gluconeogenesis pathway. In the alternative route, the formation of phosphoenolpyruvate from malate via Mdh and PckA is coupled to the oxidation of NAD^+ ^to NADH. These results suggest that plasmid-containing cells chose the gluconeogenesis pathway that generated more NADPH, which was needed by the cells for the overproduction of recombinant proteins. To our knowledge, this is the first report describing pathway alterations in recombinant *E. coli *grown on gluconeogenic media.

Two proteins involved in steps connected to PPP, ribose-5-phosphate isomerase A (RpiA) and transketolase (TktA), were down-regulated in the plasmid-containing cells but up-regulated in the host. An RpiA level time course indicated that the PPP enzyme did increase initially in response to IPTG induction to provide NADPH for cell synthesis. However, after 1 h, the level of this PPP enzyme decreased with time and reached a low level after 3 h of induction. This behavior is similar to a previous finding on the expression of rpiA, a gene coding for RpiA, which was considerably lower in *E. coli *carrying a high copy number plasmid (like pGEX-2TK in the present work) relative to *E. coli *carrying a low copy number plasmid and plasmid-free *E. coli *[25]. After a 3-h induction, the levels of the PPP-related enzymes TktA and phosphopentomutase (DeoB) decreased to a very low level in the recombinant protein overproduction strains. TktA is the key enzyme for the synthesis of aromatic amino acids and DeoB is in charge of the conversion between ribose-5-phosphate and ribose-1-phosphate. The decrease in the levels of these proteins reflects a slow-down of some cellular process.

Among proteins that were up-regulated in the host strain but down-regulated in plasmid-harboring cells, S-ribosylhomocysteinase (LuxS) is an enzyme indirectly related to protein synthesis. Through the action of LuxS on *S*-ribosylhomocysteine, the sulfur-containing amino acid homocysteine (Hcy) is produced in *E. coli *as the last intermediate in the methionine biosynthetic pathway [26]. Hcy can compete with methionine and isoleucine for the binding sites of methionyl- and isoleucyl-tRNA synthase [27]. Higher expression of LuxS can thus lead to a higher concentration of Hcy and consequently become an obstacle for the synthesis of these tRNAs. In contrast to the increase in LuxS level in the host strain, significant down-regulation of LuxS in all three plasmid-harboring bacteria released to some extent the inhibition of methionyl- and isoleucyl-tRNA synthesis and then favored protein overproduction.

### Solubility of overexpressed recombinant proteins and up-regulation of chaperones/heat shock proteins

**Figure 3:**
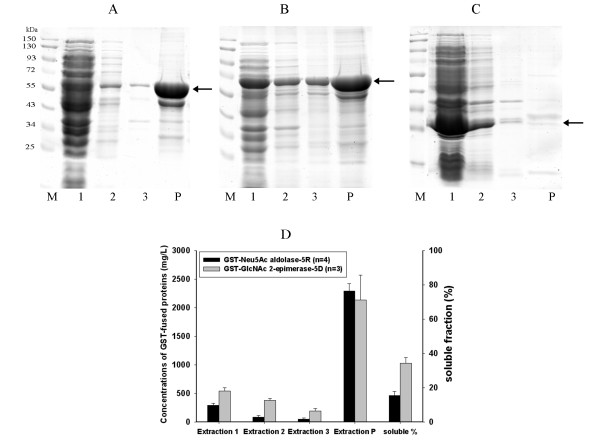
**Assay of expressed proteins by SDS-PAGE and ELISA**. SDS-PAGE of protein extracts from *E. coli *BL21 harboring pGEX-2TK-nanA-5R after 3 h of IPTG induction (A), pGEX-2TK-2ep-5D (B) and pGEX-2TK (C). Quantification of GST-fused proteins in each extraction was done by ELISA using an anti-GST antibody (D). The molecular weights of GST-Neu5Ac aldolase-5R, GST-GlcNAc 2-epimerase-5D, and GST are 59, 70 and 28 kDa, respectively, and the positions are indicated by arrows. Lane M: protein marker; lanes 1-3: the first, second and third extractions from cell pellets; lane P: extraction from aggregates as described in the Methods section. Soluble percentage was defined as the percentage of total soluble protein in extractions 1, 2 and 3 as a function of the sum of total soluble protein and insoluble protein (extraction P). Error bars stand for S.D.

Two double-tagged fusion proteins, GST-Neu5Ac aldolase-5R (535 aa) and GST-GlcNAc 2-epimerase-5D (629 aa), with molecular masses of 59 kDa and 70 kDa, respectively, were overexpressed in *E. coli *BL21. Cell pellets were disrupted in a rather small volume of lysis buffer to obtain protein fractions of repeated extraction and the insoluble fraction was recovered by completely dissolving the aggregates in high concentrations of urea with added SDS and DTT. The expression profile was revealed by SDS-PAGE and ELISA (Figure 3). As shown in Figure 3, with a limited volume of lysis buffer, most soluble recombinant proteins were recovered in the first extraction and protein concentration decreased gradually in the second and third extractions. The concentration of GST-Neu5Ac aldolase-5R in extractions 1, 2, 3, and P was 287.4, 81.5, 50.5, and 2289.7 μg/mL, respectively; the GST-GlcNAc 2-epimerase-5D concentration in those extractions was 538.5, 373.4, 190.5, 2136.9 μg/mL, respectively. Fusion proteins collected in extractions 1, 2 and 3 were grouped together as the soluble faction, whereas the fusion proteins recovered in extraction P were regarded as insoluble. The soluble percentage of GST-GlcNAc 2-epimerase-5D was 36.1 wt%, which was more than twice that of GST-Neu5Ac aldolase-5R (15.4 wt%). Our previous study showed that both GST-GlcNAc 2-epimerase-5D and GST-Neu5Ac aldolase-5R collected in P fractions were enzymatically active [5]. Thus, a high proportion of recombinant protein possessing enzymatic activity was observed in the insoluble form in both bacteria.

**Figure 4:**
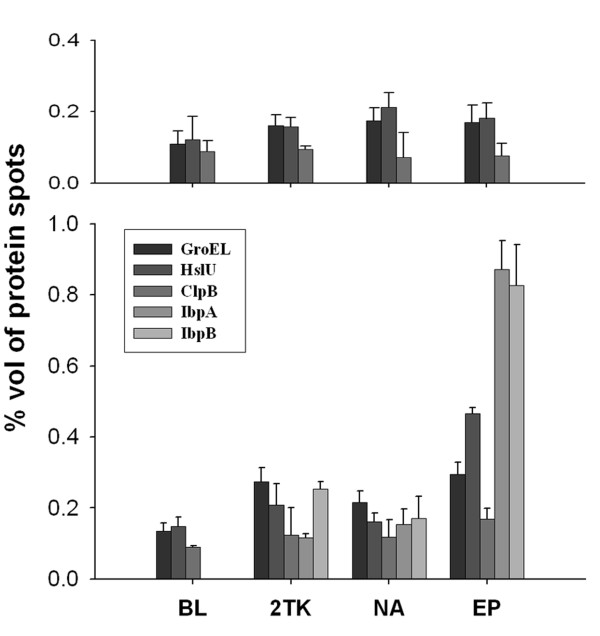
**Expression of heat shock proteins in the host and recombinant *E. coli***. Expression levels of heat shock proteins prior to induction (upper panel) and at 3 h post-induction (lower panel) in the host strain and recombinant protein expressing strains. Abbreviations are as follows: **BL**, *E. coli *BL21; **2TK**, *E. coli *BL21 harboring pGEX-2TK; **NA**, *E. coli *BL21 harboring pGEX-2TK-nanA-5R; **EP**, *E. coli *BL21 harboring pGEX-2TK-2ep-5D. The % volume was defined as the value of the intensity integration over the feature area of one spot divided by the total intensity integration over all of the spots in the whole gel image (100%). Error bars stand for S.D.

The solubility of overexpressed proteins could be correlated well to the expression level of cellular chaperones/heat shock proteins. Figure 4 shows the expression level of five chaperones/heat shock proteins, GroEL, ClpB, HslU, IbpA and IbpB, in the host strain and recombinant plasmid-harboring cells after a 3-h induction with IPTG. Comparing the 2-DE maps of cellular proteins for pGEX-2TK-nanA-5R-harboring *E. coli *BL21 and pGEX-2TK-2ep-5D- harboring *E. coli *BL21 harvested at 0 h and 3 h post-induction without IPTG (Figure 1 and Additional file 2), we found that the expression levels of these heat shock proteins (ClpB, GroEL, IbpA, IbpB and HslU) at T3 were almost identical to that at T0 in the recombinant plasmid-harboring cells. The changes in the expression level of heat shock proteins in the presence of IPTG were thus mainly due to the overexpression of recombinant proteins. According to a proposed mechanism, disaggregation of overexpressed protein in *E. coli *is carried out by a network of ATPase chaperones consisting of a DnaK core assisted by the cochaperones DnaJ, GrpE, ClpB and GroEL-GroES [28]. ClpB plays a starting role in the sequential mechanism of disaggregation by interacting with aggregates. In the final step of the disaggregation process, GroEL and DnaK complete the refolding of solubilized polypeptide chains into native proteins. Our results indicate that after the induction of plasmid-encoded proteins for overexpression, three chaperone proteins (ClpB, GroEL and HslU) were up-regulated, while the change of these protein levels in the host strain was not significant. However, the degree of up-regulation of these proteins was different among the three plasmid-containing strains. The intensity of up-regulation followed this order: pGEX-2TK-2ep-5D- > pGEX-2TK- > pGEX-2TK-nanA-5R-harboring cells. ClpB was significantly up-regulated in the pGEX-2TK-2ep-5D-harboring cells because of the formation of protein aggregates during the overproduction of recombinant protein, but the alteration of ClpB level in the cells harboring pGEX-2TK was insignificant since the plasmid-encoded protein (GST) was almost fully soluble. Similarly to ClpB expression, the level of GroEL in pGEX-2TK-2ep-5D-harboring cells was also significantly up-regulated. These results could well explain why the overexpressed fusion protein GST-GlcNAc 2-epimerase-5D was more soluble than the double-tagged fusion protein GST-Neu5Ac aldolase-5R. Likewise, the up-regulation of GroEL in pGEX-2TK cells could help the solubilization of plasmid-encoded protein GST.

The expression of HslU in recombinant bacteria in response to IPTG induction was very similar to that of ClpB. Both HslU (also named ClpY) and ClpB are members of the Clp/Hsp100 chaperone family. HslU is a chaperone subunit of a proteasome-like degradation complex. It typically forms a complex with HslV which functions as an ATP-dependent protease [29]. The HslV portion functions as a protease and the HslU is an ATPase. The complex directs ATP-dependent deoligomerization and degradation of substrate proteins. Like ClpB, the promoter for HslU-HslV is also recognized by σ^32^. HslU was induced to express with ClpB for the disaggregation of inclusion bodies produced in plasmid-harboring cells, especially in the pGEX-2TK-2ep-5D harboring cells.

Two small heat shock proteins, IbpA and IbpB, were produced only with the overexpression of plasmid-coded proteins, and their expression levels increased with induction time. IbpA/IbpB are tightly associated with inclusion bodies formed during heterologous protein production in *E. coli *cells [30]. In protein aggregates, IbpA/IbpB proteins bind partially folded proteins until disaggregating chaperone ClpB becomes available [31]. Since the pGEX-2TK-encoded protein GST was almost fully soluble, ClpB was not induced to up-regulation by the overexpression of this heterologous protein. The expression of IbpA/IbpB in the GST-overexpressing cells was thus at a relatively low level in comparison with the cells overexpressing GST-GlcNAc 2-epimerase-5D. In the GST-overexpressing cells, IbpA and IbpB were induced to help to bind the nascent polypeptides and prevent them from aggregates.

Neu5Ac aldolase and GlcNAc 2-epimerase here were expressed in the form of double-tagged proteins. The GST tag fused to the N-terminus of the protein of interest allows the fusion protein to be purified to near homogeneity by affinity method. However, the tag may alter protein conformation or affect biologically important functions. Since a linker sequence and a protease cleavage site are built between the tag and the target protein within the expression vector, these shortcomings can be overcome and the tag can be removed after purification if necessary. In the present study, both GST and the polyionic tag (5D or 5R) were soluble, but the overexpressed double-tagged fusion proteins were partially soluble as shown in Figure 3. The addition of polyionic tag did not influence the expression pattern of fusion protein. As shown in Additional file 4, the solubility of GST-fused Neu5Ac aldolase was not changed by the presence of 5R tag. Even with the GST as soluble partners, the insoluble fractions of these two recombinant proteins made up a large amount (more than 60%) of the total recombinant proteins. This result suggests that recombinant proteins exist in different conformational states ranging from insoluble forms to soluble forms, which is in accordance with previous observations [32]. In particular, GST-Neu5Ac aldolase-5R had a very small amount of soluble recombinant protein in the second and third extractions. Comparing these results with the expression profile of heat shock proteins in *E. coli *BL21 producing GST-Neu5Ac aldolase-5R, all of them went up to a higher level in the strain producing GST-GlcNAc 2 epimerase-5D, which exhibited higher protein amount in the 1st, 2nd, and 3rd extractions. The gene product of the blank plasmid (pGEX-2TK), GST was almost totally soluble (Figure 3), and just a trace of protein could be detected in the 2nd, 3rd and P fractions. The levels of heat shock proteins expressed in *E. coli *BL21 producing GST in Figure 4 show that GroEL was induced to an extent close to that in the GST-2 epimerase-5D-producing cells, whereas ClpB and IbpA were expressed at a level as low as in the GST-nanA-5R-producing cells. Taken together, these results indicate that expressed GST (in pGEX-2TK-harboring cells), as a solubility enhancer, mainly exists in native states under the demand of GroEL, indicating that the trigger of disaggregating chaperones and small heat shock proteins is not crucial when only a small amount of insoluble protein is present.

### Expression level of heat shock proteins and sigma factor 32

**Figure 5:**
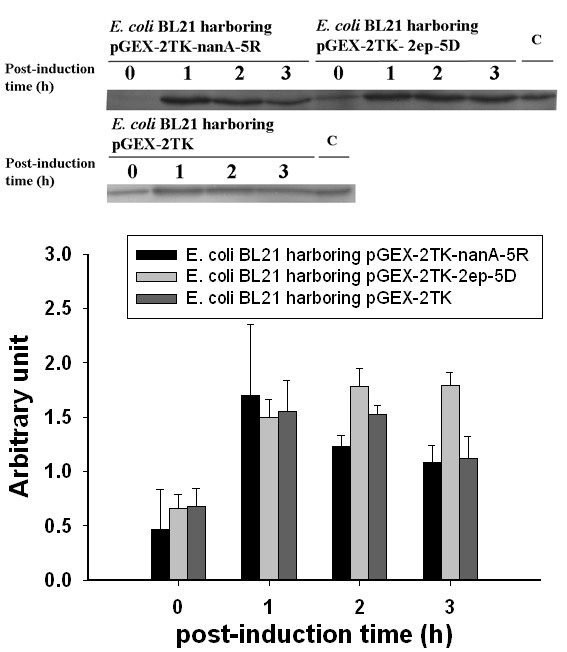
**Western blot analysis of sigma 32 in recombinant protein expressing strains**. Western analyses were performed for sigma 32 expressions in GST-, GST-GlcNAc 2-epimerase-5D-, and GST-Neu5Ac aldolase-5R-expressing *E. coli *BL 21 induced for 0, 1, 2, and 3 h. The samples marked with C (in upper panel) referre to the protein mixture loaded in each gel to serve as the inter-membrane control. In the graphical plot, the arbitrary unit was calculated by dividing the image intensity of the anti-σ^32 ^band on developed film by that of the identical control on the same film. Error bars stand for S.D. (n = 3).

Heat shock proteins IbpA and IbpB, HslU, and ClpB are all σ^32^-dependent heat shock proteins, whereas GroEL can bind σ^32 ^to regulate its activity [33]. The σ^32 ^in *E. coli*, encoded by the rpoH gene, is a transcription factor enabling RNA polymerase to recognize the promoter of heat shock proteins. The expression levels of σ^32 ^in the recombinant strains as determined by western blot are compared in Figure 5. The results indicate that the expression levels of σ^32 ^just before induction were comparable in all strains. In the following three hours of IPTG induction, σ^32 ^reached a maximum (1.67 AU) at 1 h and decreased to 1.08 AU at 3 h post-induction in the GST-Neu5Ac aldolase-5R-expressing strain. Because of this decrease in σ^32 ^level, the accumulation of aggregated GST-Neu5Ac aldolase-5R seemed not to induce the heat shock proteins essential for the disaggregation process in this study.

In the GST-GlcNAc 2-epimerase-5D expressing cells, the σ^32 ^level increased gradually to 1.8 AU (arbitrary unit) and remained at the higher value. This explains why the proteins IbpA, IbpB, HslU and ClpB were all up-regulated after a 3-h induction. The up-regulation of these heat shock proteins due to the sustained expression of σ^32 ^promotes the solubility of recombinant proteins and helps rescue their active conformation. On the other hand, the response of σ^32 ^was relatively lower in *E. coli *BL21 harboring pGEX-2TK, which was in agreement with the expression level of the heat shock proteins. The expression profile of σ^32 ^in *E. coli *producing GST-Neu5Ac aldolase-5R was similar to that in cells carrying plasmid pGEX-2TK, exhibiting a tendency to decline after the first hour of induction, leading to a lower chaperone/heat shock protein expression level. Thus, the aggregated GST-Neu5Ac aldolase-5R proteins were still trapped in the insoluble fraction under conditions of low heat shock response.

Although amplification of the genes encoding IbpA and IbpB could enhance the production of recombinant proteins, overexpression of IbpA and IbpB resulted in more aggregate (inclusion bodies) than soluble protein [18]. A previous study revealed that co-expression of heterologous proteins with a four-chaperone system (GroEL-GroES, DnaK-DnaJ-GrpE, ClpB and IbpA/IbpB) led to a remarkable increase in the solubility of various recombinant proteins [9]. These results suggest that these σ^32 ^-regulated chaperones work together in a cooperative way. In many cases, bacterial inclusion bodies are formed by highly functional enzymatic forms and the solubility of the expressed proteins does not parallel conformational quality [10]. This is also true in the present work with the overexpression of double-tagged fusion proteins of GlcNAc 2-epimerase and Neu5Ac aldolase [5]. Because the protease-based heat shock protein HslU was up-regulated along with ClpB, we agree with the conclusion of Garcia-Fruitos et al. that the *E. coli *quality control system (composed of cytosolic chaperones and proteases) promotes protein solubility instead of conformational quality through over-committed proteolysis of aggregation-prone polypeptides, irrespective of their conformational status and biological properties [10]. In summary, maintaining the expression of σ^32 ^at a high level could be a useful strategy for promoting the solubility of overexpressed proteins and misfolded polypeptides that restore their biological activity to some extent via enhanced expression of cytosolic chaperones.

## Conclusions

Proteome profiles in host and recombinant strains were altered in the presence of the gene expression inducer IPTG. The induction for overexpression of plasmid-encoded proteins caused generally low expression of cellular proteins and down-regulation of enzymes in charge of steroid and amino acid synthesis. Proteins related to the pentose phosphate pathway were also down-regulated in plasmid-harboring cells. Most interestingly, different expression patterns of proteins in the TCA cycle and the gluconeogenesis pathway were found between host and recombinant cells. When cultured in LB medium, host cells underwent gluconeogenesis likely via Mdh and PckA, whereas in the plasmid-harboring cells, gluconeogenesis occurred mainly through MaeB, coupling it to the generation of NADPH for cell biosynthesis. Also, the homocysteine-producing enzyme LuxS, which at higher levels can block t-RNA synthesis, was down-regulated upon the overexpression of plasmid-encoding proteins. Even when the recombinant proteins were overexpressed with soluble tags, (such as GST and polyionic peptide 5R or 5D), most of the overexpressed protein was in the insoluble form. After IPTG induction, chaperones/heat shock proteins, including ClpB, HslU, GroEL, IbpA and IbpB, were up-regulated to varying extents among the strains. The disaggregation chaperone ClpB and HslU were significantly up-regulated for the dissolution of inclusion bodies produced in the pGEX-2TK-2ep-5D-harboring cells. The up-regulation of ClpB was insignificant in the pGEX-2TK-harboring cells since overexpressed GST was fully soluble. In contrast to the pGEX-2TK-2ep-5D-harboring strain, the up-regulation of ClpB and other chaperones/heat shock proteins in the pGEX-2TK-nanA-5R-harboring strain was relatively insignificant, and the soluble fraction of overexpressed protein was lower in the latter. The solubility of overexpressed protein thus correlated well with the expression of these chaperones/heat shock proteins. Furthermore, the expression of soluble protein was enhanced by the up-regulation of ClpB, HslU, IbpA, and IbpB under control of σ^32^-recognized promoters in the plasmid-harboring *E. coli *strains. In the double-tagged GlcNAc 2-epimerase-expressing cells, the expression of σ^32 ^remained at a higher level and even increased slightly with induction time, while the σ^32 ^level in double-tagged Neu5Ac aldolase-expressing cells dropped after reaching a peak value at 1 h post-induction. Sustained expression of σ^32 ^at a higher level during the overexpression of recombinant proteins could be crucial to promoting the solubility of overexpressed proteins.

## Methods

### Bacterial strains, plasmids and growth conditions

The bacteria used in this study are the host *E. coli *BL21 and three plasmid-harboring *E. coli *BL21 that can respectively overexpressing GST, GST-Neu5Ac-aldolase-(arginine)_5_, and GST-GlcNAc 2-epimerase-(aspartate)_5 _[5]. Gene sources of Neu5Ac aldolase and GlcNAc 2-epimerase were from *E. coli *K12 and *Synechocystis *sp. strain PCC6803, respectively. Flask cultures (400 mL of culture in each 1-L flask) were carried out at 28°C and 150 rpm in LB medium (1% tryptone, 1% NaCl and 0.5% yeast extract). Ampicillin was added at a final concentration of 100 μg/mL to culture of strains harboring plasmids. Cell growth was monitored by measuring the value of OD_600 _using a Beckman DU-640 spectrophotometer (Beckman, Fullerton, CA). Upon reaching OD_600 _~ 0.8, induction of recombinant proteins was started by adding IPTG at a final concentration of 1 mM and cells were further cultivated for three hours. Cells were then harvested by centrifugation (5857 × g, 15 min, 4°C) and the resulting pellets were stored at -20°C until further use.

### SDS-PAGE and solubility analysis

Collected cell pellets were resuspended in a lysis buffer containing 100 mM NaCl, 50 mM Na_2_HPO_4_, 0.1 mM EDTA, 10 mM β-mercaptoethanol, 0.2% Triton X-100, 25 μg/L PMSF and 40 μg/L lysozyme at a 1:33 volume ratio of lysis buffer to bacterial broth [5]. Cells were lysed with a probe sonicator XL-2020 (Misonix) in an ice bath for 15 min and then centrifuged at 7650 × g for 15 min at 4°C. The supernatant was collected as the first protein extract and the precipitate was treated twice by the same procedure to get the second and third protein extracts. The remaining aggregates was dissolved in 8 M urea, 4% SDS and 1% DTT and centrifuged at 12,000 × g for 2 min at room temperature. The resulting supernatant was denoted as extraction P. All protein extracts were stored at -80°C in aliquots. SDS-PAGE was carried out on a 10% gel and bands were visualized by Coomassie blue staining. The concentration of GST-fused protein in each fraction was determined by using a One-Step ELISA™ GST Detection kit (GenScript, Piscataway, NJ).

### Activity assay

The activity of GST-GlcNAc 2-epimerase-5D was assayed based on the formation of N-acetyl-D-mannosamine (ManNAc) from N-acetyl-D-glucosamine (GlcNAc). By incubating the protein preparation with 1 ml of assay solution containing 100 mM GlcNAc, 10 mM MgCl_2_, 5 mM ATP in 100 mM Tris-HCl buffer (pH 7.5) for 10 min at 37°C. The reaction was then stopped by heating and the amount of ManNAc produced was determined by HPLC using the Aminex-87H column [5]. The activity of GST-Neu5Ac aldolase-5R was determined using Neu5Ac as the substrate and the decrease of Neu5Ac was estimated. The protein preparation was incubated with 1 ml of Tris-HCl buffer (100 mM, pH 7.5) containing 20 mM Neu5Ac for 10 min at 37°C. The reaction was then stopped by heating and the amount of Neu5Ac consumed was determined by HPLC using the Aminex-87H column [5].

### 2-DE and image analysis

Cell pellets were washed four times in low salt washing buffer (3 mM KCl, 1.5 mM KH_2_PO_4_, 68 mM NaCl and 9 mM NaH_2_PO_4_). Washed pellets were resuspended in lysis buffer consisting of 8 M urea, 4% CHAPS and 1% DTT and then disrupted by short bursts of sonication. The cell lysate was clarified by centrifugation (13,000 × g, 30 min, 15°C) and the clear supernatant was stored at -80°C in aliquots. Protein quantification was performed using BioRad protein assay reagent and bovine serum albumin was used as the protein standard.

A 40 μg aliquot of protein extract from host cells (or 48 μg of protein extract from recombinant protein expressing cells) was mixed with rehydration buffer (8 M urea, 2% CHAPS, 1% DTT and 0.2% BioLyte 3-10) to make a final volume of 340 μL and then loaded onto a pH 4-7,18-cm Immobiline DryStrip (GE Healthcare, Fairfield, CT). The loaded strip was then in-gel rehydrated for 16 h at 20°C under 50 V. Proteins underwent isoelectric focusing in a Protean IEF Cell (BioRad, Hercules, CA) programmed as follows: 300 V for 1 h; 1000 V for 1 h; 1000 to 8000 V within 3 h, and then kept at 8000 V until a total voltage-hour of 65 kVh was reached. After isoelectric focusing, strips were equilibrated in two sequential equilibrium buffers containing 2% (w/v) DTT and 2.5% (w/v) iodoacetamide for 15 min. Electrophoresis in the second dimension was carried out on a 12.5% SDS-polyacrylamide gel in a Protean II xi Cell (BioRad, Hercules, CA). Three replicates were performed in this study. Gels were then stained as described [34] with a modification consisting of the reduction of the concentration of silver nitrate to 0.2% (w/v).

Stained gels were scanned on an ImageScanner densitometer (GE Healthcare, Fairfield, CT) at 300 dpi resolution with a blue filter and images were analyzed by ImageMaster 2D platinum software (version 5, GE Healthcare, Fairfield, CT) [35]. For each protein spot, the spot outline was determined by setting parameters for the software as smooth: 4, min area: 9, saliency: 300 in the spot detection function. The software then computed spot feature area and spot volume automatically once the outline had been decided. The spot volume is defined as the value of the image intensity integration over the feature area of one spot. The relative spot volume (% volume) was calculated using the following formula: % volume = the volume of one spot divided by the sum of the volumes of all spots in a gel. Differences in % volume for each spot between groups were evaluated using *t*-tests. All statistical analyses were performed using SPSS software. Protein spots showing high differential expression and with *p *< 0.05 were given priority for identification.

### In-gel digestion

Protein spots of interest were sliced from silver-stained gels followed by in-gel digestion as described [36]. Home-made StageTip [37] was used to remove salts from the extracted solution. The eluate was evaporated to dryness under vacuum and stored at -20°C for further analysis.

### Protein identification by MALDI MS and MS/MS

The dried sample was resuspended in 50% acetonitrile and 0.1% formic acid and then mixed 1:1 with matrix solution consisting of 5 mg/mL α-cyano-4-hydroxycinnamic acid (CHCA) in 50% acetonitrile, 0.1% v/v TFA and 2% w/v ammonium citrate. The mixture was spotted onto the 96-well format MALDI sample plate. Data directed acquisition on the Q-TOF Ultima™ MALDI instrument was fully automated. Within each well, all parent ions meeting the predefined criteria (any peak within the *m/z *800-3000 range with intensity above 10 count ± include/exclude list) were selected for CID MS/MS using argon as collision gas and a mass dependent ± 5V rolling collision energy. The instrument was externally calibrated to less than 5 ppm accuracy over the mass range of *m/z *800 - 3000 and further adjusted with Glu-Fibrinopeptide B as the near-point lock mass calibrant during data processing. MS and MS/MS survey were processed using Micromass ProteinLynx™ Global Server (PGS) 2.0 data processing software. The output ".txt" files for peptide mass fingerprinting (PMF) and ".pkl" files for peptide fragment fingerprinting (PPF) were searched against the NCBI database using the Mascot program.

### Western blot analysis

One mL of broth was collected every hour from the time of induction and centrifuged at 7267 × g to obtain the cell pellet. The pellet was resuspended in SDS sample buffer with a volume equivalent to the value of OD_600 _× 0.8 mL and the mixture was boiled for 10 min. Equal volumes of the clarified supernatants after centrifugation at 10,464 × g were loaded onto a 12.5% SDS-polyacrylamide gel. After SDS-PAGE, proteins were blotted onto PVDF membranes (GE Healthcare, Fairfield, CT) following the Complete Mini-Genie Blotter (Idea Scientific Company, Minneapolis, MN) instructions. The PVDF membrane was blocked in 5% nonfat dry milk in TBST (20 mM Tris-HCl, 500 mM NaCl and 0.05% Tween 20) for 2 h at room temperature. The washed blot was then incubated with 1:1000 dilution of anti-*E. coli *σ^32 ^factor monoclonal antibody (NeoClone, Madison, WI) overnight at 4°C. HRP-conjugated rabbit anti-mouse IgG polyclonal antibody (Abcam, Cambridge, MA) was used as the secondary antibody, at a dilution of 1:3000 and incubated with the blot for 1 h at room temperature. Bands of σ^32 ^factor were detected using Novex^® ^ECL Chemiluminescent Reagent Kit (Invitrogen, Carlsbad, CA). Developed films were scanned and analyzed as described in the 2-DE and Image Analysis section. In every transblotting membrane the same protein mixture was loaded together with the samples in other lane as the inter-membrane control. Image intensities of the anti-σ^32 ^band on developed film were normalized to that of this identical control on the same film.

## Competing interests

The authors declare that they have no competing interests.

## Authors' contributions

CHC carried out all experimental works and WCL was in charge of planning and conducting the study, and writing the manuscript. All authors read and approved the final manuscript.

## Supplementary Material

Additional file 1 **Supplemental Figure 1: Growth curves of host and recombinant *E. coli *BL21**. Bacteria were cultivated in LB medium at 28°C. The arrow indicates the addition of IPTG. Click here for file

Additional file 2 **Supplemental Figure 2: Representative 2-DE gels of *E. coli *BL21 host and plasmid-bearing strains**. Two-dimensional electrophoresis (2-DE) of the lysate of *E. coli *BL21 cells (A, B) and pGEX-2TK-harboring (C, D) *E. coli *BL21 cells harvested before (A, C) and after 3-h IPTG induction (B, D). Subfigures E, F, and G are 2-DE of the lysate of *E. coli *BL21, pGEX-2TK-nanA-5R-harboring *E. coli *BL21, and pGEX-2TK-2ep-5D- harboring *E. coli *BL21, respectively, harvested at 3 h post-induction without IPTG. Spot indicated by an arrow in subfigure D was the recombinant GST protein. Click here for file

Additional file 3 **Supplemental Figure 3: Time courses of protein expression**. Time courses of the expression levels of differentially expressed proteins in *E. coli *BL21 (solid lines and circles) and *E. coli *BL21 harboring pGEX-2TK-2ep-5D (dashed lines and open circles). Click here for file

Additional file 4 **Supplemental Figure 4: Assay of tagged proteins by SDS-PAGE**. SDS-PAGE of protein extracts from *E. coli *BL21 overexpressing double-tagged GST-Neu5Ac aldolase-5R (A) and single-tagged GST-Neu5Ac aldolase (B) after 3 h of IPTG induction. Lane M: protein marker; lanes 1-3: the first, second and third extractions from cell pellets; lane P: extraction from aggregates as described in the Methods section. The double-tagged GST-Neu5Ac aldolase-5R was expressed in bacteria harboring pGEX-2TK-nanA-5R; while the single-tagged GST-Neu5Ac aldolase was expressed in bacteria harboring plasmid pGEX-1λT with an inserted sequence coding for Neu5Ac aldolase. Arrows indicate double-tagged GST-Neu5Ac aldolase-5R (in A) and single-tagged GST-Neu5Ac aldolase (in B). Click here for file
