# Supplementary material for: Protein solubility and differential proteomic profiling of recombinant Escherichia coli overexpressing double-tagged fusion proteins
Source: Microb Cell Fact. 2010 Aug 28;9:63. doi: 10.1186/1475-2859-9-63 (PMC2940792; doi:10.1186/1475-2859-9-63)

Supplemental Figure 1: Growth curves of host and recombinant *E. coli* BL21. Bacteria were cultivated in LB medium at 28°C. The arrow indicates the addition of IPTG.

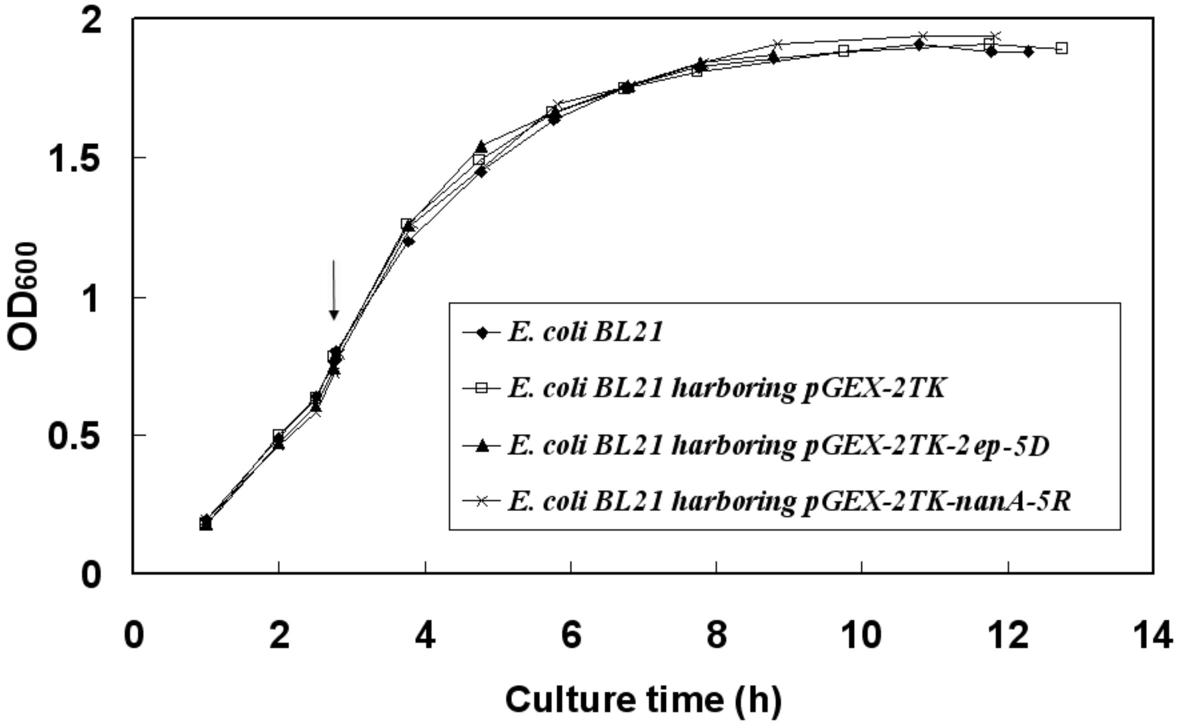

Supplement: Additional file 1 — Supplemental Figure 1: Growth curves of host and recombinant E. coli BL21. Bacteria were cultivated in LB medium at 28°C. The arrow indicates the addition of IPTG. [file 1475-2859-9-63-S1.PDF]
