# Supplementary material for: Protein solubility and differential proteomic profiling of recombinant Escherichia coli overexpressing double-tagged fusion proteins
Source: Microb Cell Fact. 2010 Aug 28;9:63. doi: 10.1186/1475-2859-9-63 (PMC2940792; doi:10.1186/1475-2859-9-63)
Supplement: Additional file 2 — Supplemental Figure 2: Representative 2-DE gels of E. coli BL21 host and plasmid-bearing strains. Two-dimensional electrophoresis (2-DE) of the lysate of E. coli BL21 cells (A, B) and pGEX-2TK-harboring (C, D) E. coli BL21 cells harvested before (A, C) and after 3-h IPTG induction (B, D). Subfigures E, F, and G are 2-DE of the lysate of E. coli BL21, pGEX-2TK-nanA-5R-harboring E. coli BL21, and pGEX-2TK-2ep-5D- harboring E. coli BL21, respectively, harvested at 3 h post-induction without IPTG. Spot indicated by an arrow in subfigure D was the recombinant GST protein. [file 1475-2859-9-63-S2.PDF]

Supplemental Figure 2: Representative 2-DE gels of *E. coli* BL21 host and plasmid-bearing strains.

Two-dimensional electrophoresis (2-DE) of the lysate of *E. coli* BL21 cells (A, B) and pGEX-2TK-harboring (C, D) *E. coli* BL21 cells harvested before (A, C) and after 3-h IPTG induction (B, D). Subfigures E, F, and G are 2-DE of the lysate of *E. coli* BL21, pGEX-2TK-nanA-5R-harboring *E. coli* BL21, and pGEX-2TK-2ep-5D-harboring *E. coli* BL21, respectively, harvested at 3 h post-induction without IPTG. Spot indicated by an arrow in D was the recombinant GST protein.

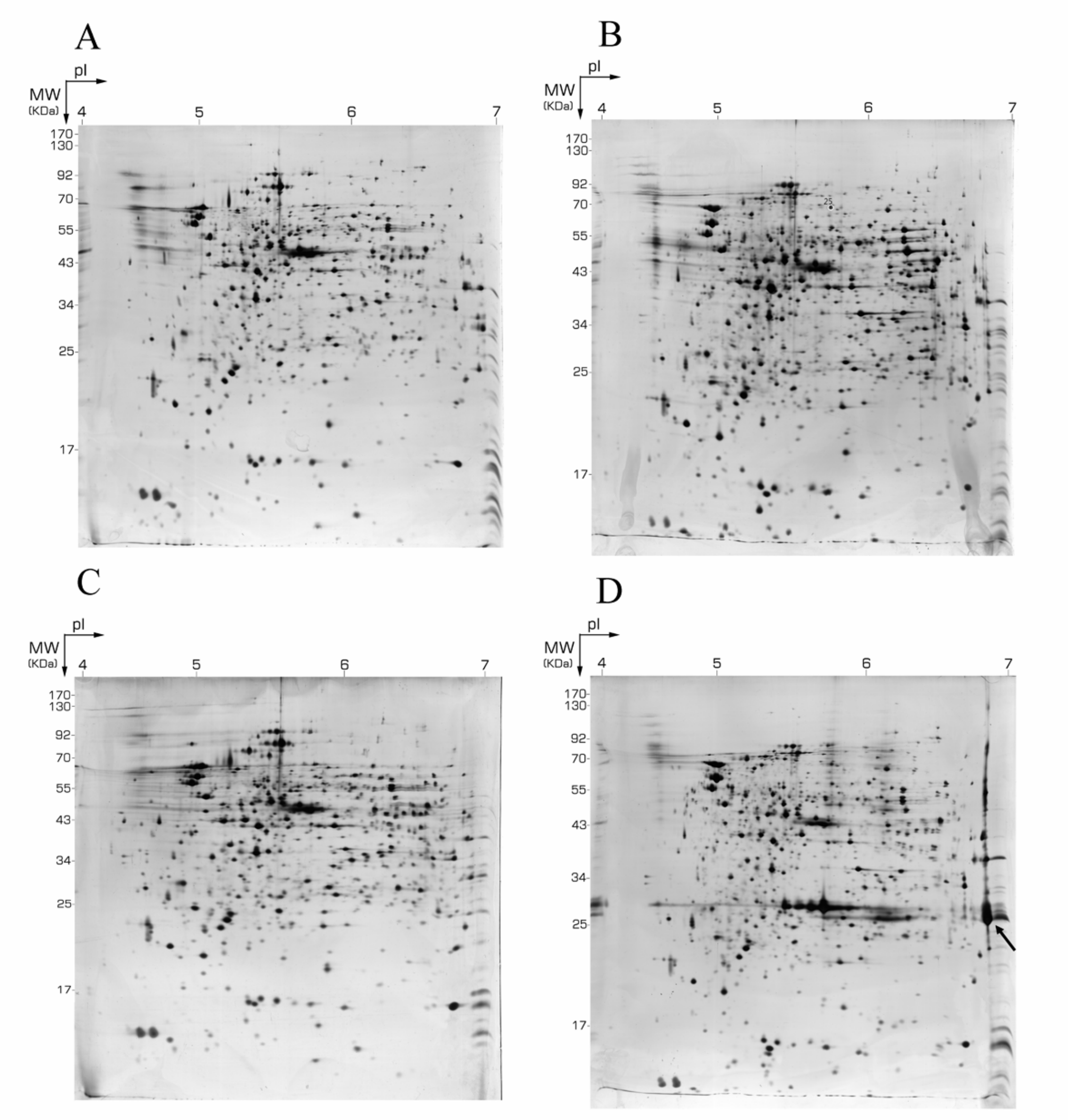

E

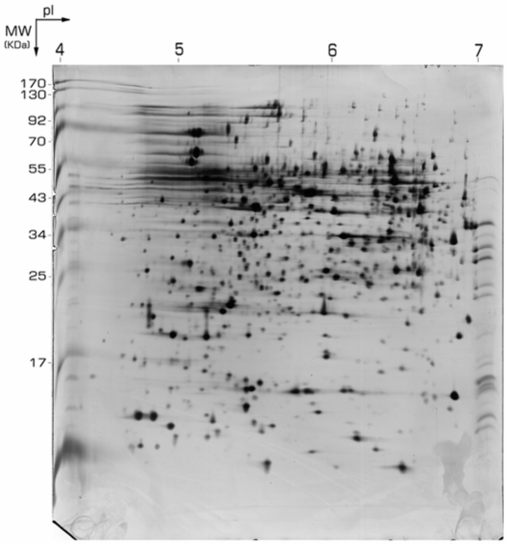

MW
(KDa)
pI
4
5
6
7
170
130
92
70
55
43
34
25
17

F

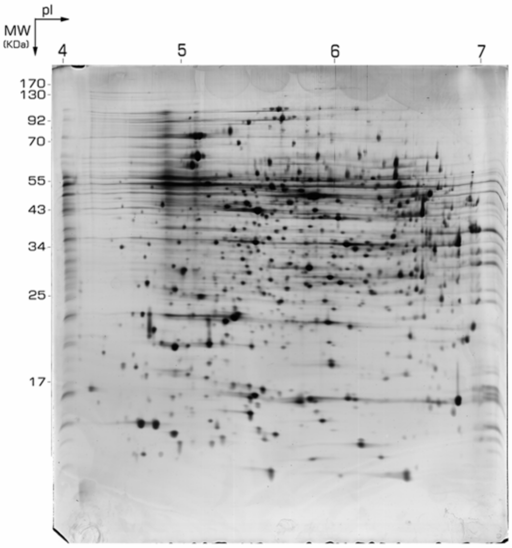

MW
(KDa)
pI
4
5
6
7
170
130
92
70
55
43
34
25
17

G

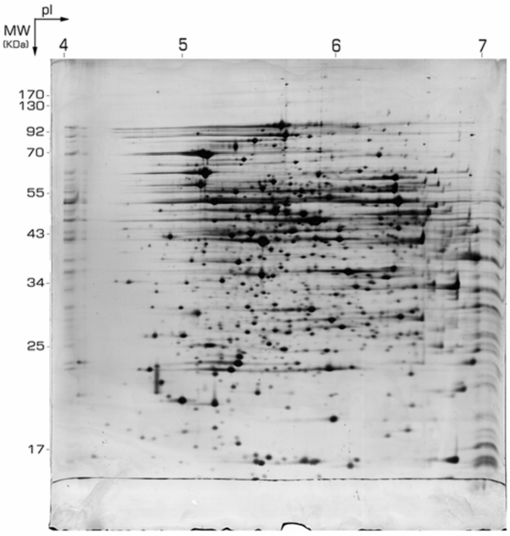

MW
(KDa)
pI
4
5
6
7
170
130
92
70
55
43
34
25
17
