# Supplementary material for: Protein solubility and differential proteomic profiling of recombinant Escherichia coli overexpressing double-tagged fusion proteins
Source: Microb Cell Fact. 2010 Aug 28;9:63. doi: 10.1186/1475-2859-9-63 (PMC2940792; doi:10.1186/1475-2859-9-63)
Supplement: Additional file 3 — Supplemental Figure 3: Time courses of protein expression. Time courses of the expression levels of differentially expressed proteins in E. coli BL21 (solid lines and circles) and E. coli BL21 harboring pGEX-2TK-2ep-5D (dashed lines and open circles). [file 1475-2859-9-63-S3.PDF]

Supplemental Figure 3: Time courses of protein expression. Time courses of the expression levels of differentially expressed proteins in *E. coli* BL21 (solid lines and circles) and *E. coli* BL21 harboring pGEX-2TK-2ep-5D (dashed lines and open circles).

Heat shock protein (1-5)

1.

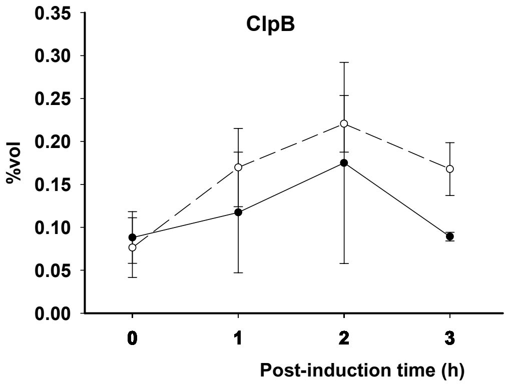

2.

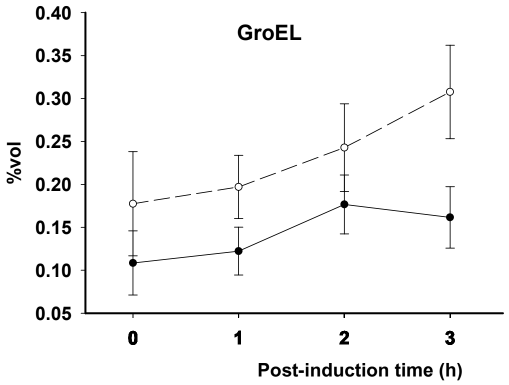

3.

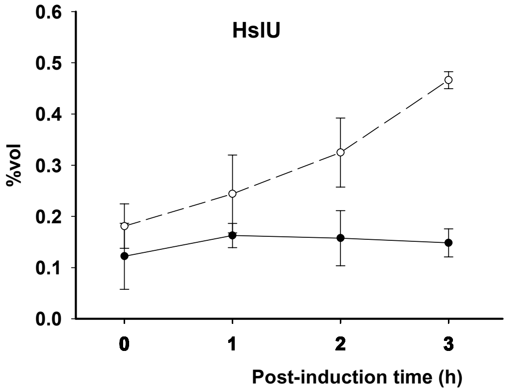

4.

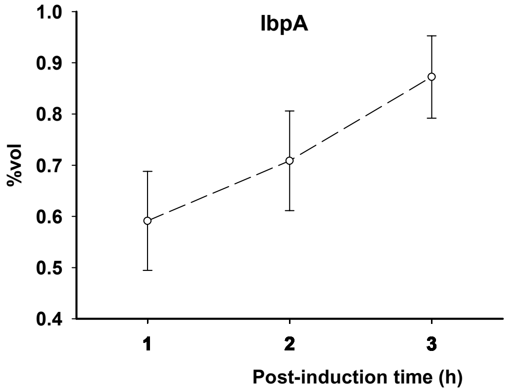

5.

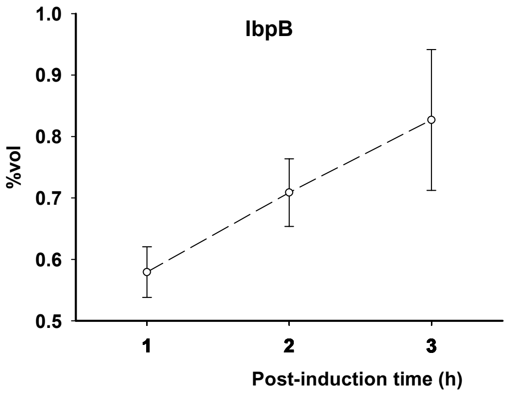

Central metabolism and TCA cycle (6-12)

6.

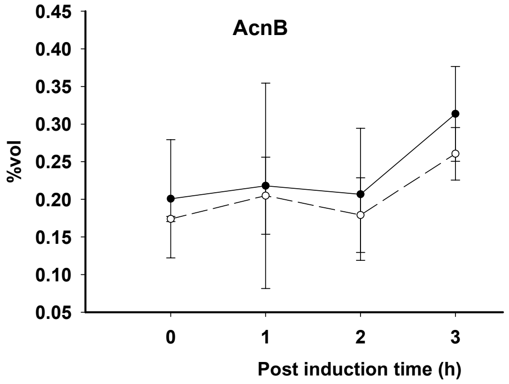

7.

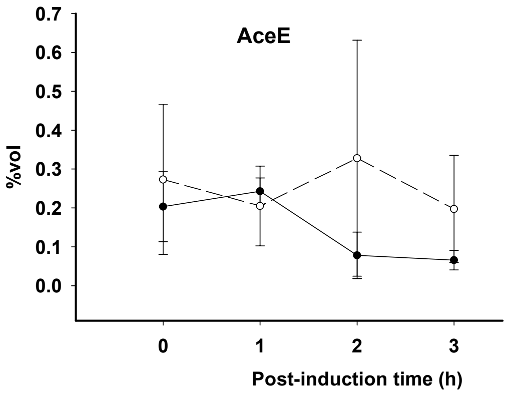
AceE
%vol
0.0
0.1
0.2
0.3
0.4
0.5
0.6
0.7
0
1
2
3
Post-induction time (h)

8.

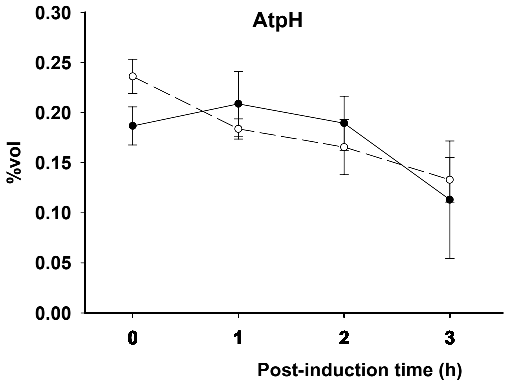
AtpH
%vol
0.00
0.05
0.10
0.15
0.20
0.25
0.30
0
1
2
3
Post-induction time (h)

9.

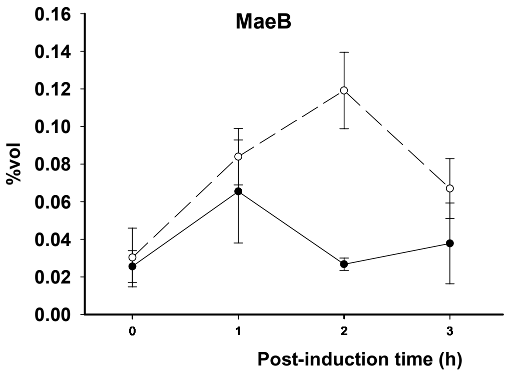
MaeB
%vol
0.00
0.02
0.04
0.06
0.08
0.10
0.12
0.14
0.16
0
1
2
3
Post-induction time (h)

10.

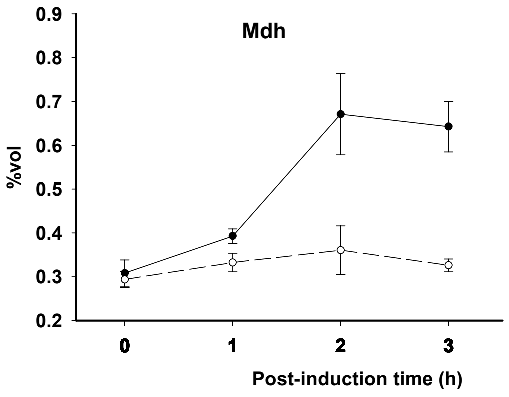
Mdh
%vol
0.2
0.3
0.4
0.5
0.6
0.7
0.8
0.9
0
1
2
3
Post-induction time (h)

11.

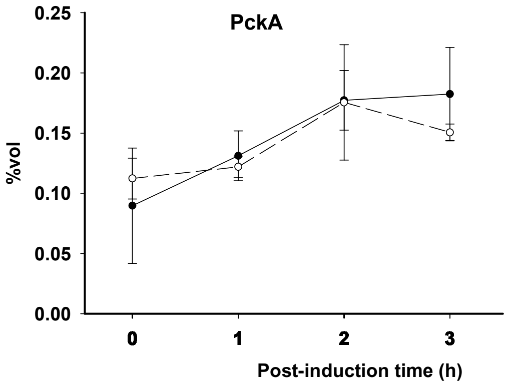
PckA
%vol
0.00
0.05
0.10
0.15
0.20
0.25
0
1
2
3
Post-induction time (h)

12.

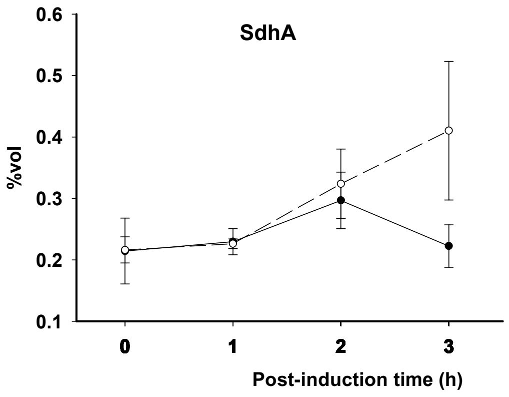
SdhA
%vol
0.1
0.2
0.3
0.4
0.5
0.6
0
1
2
3
Post-induction time (h)

Nucleotide and nucleoside metabolic process (13-19)

13.

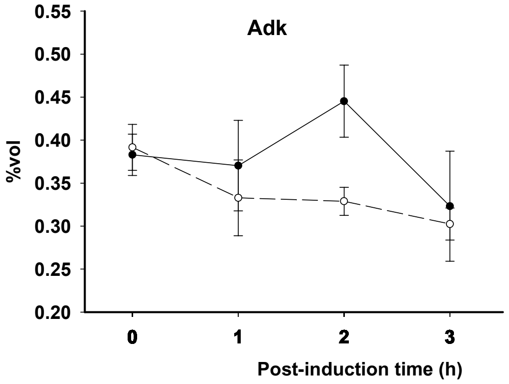

14.

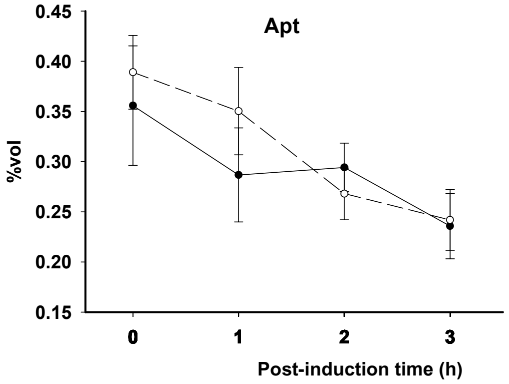

15.

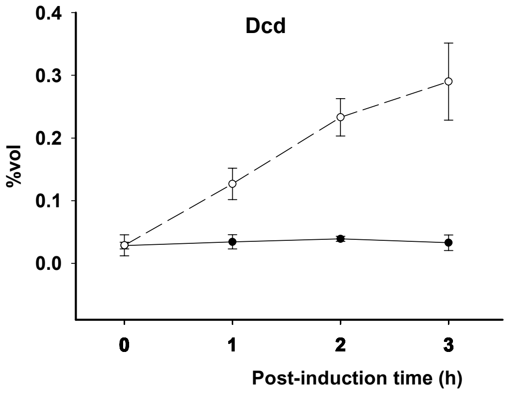

16.

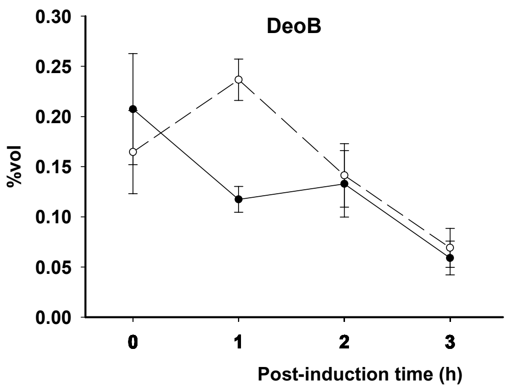

17.

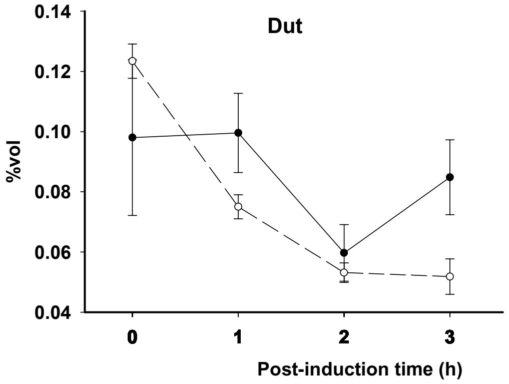

18.

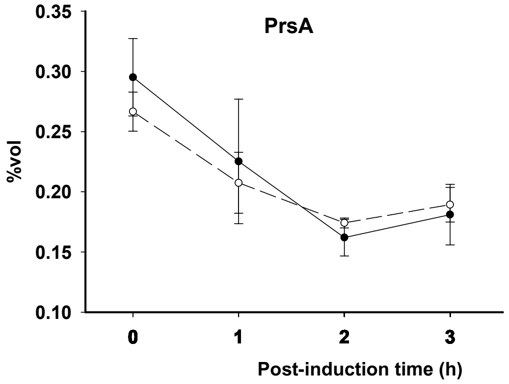

19.

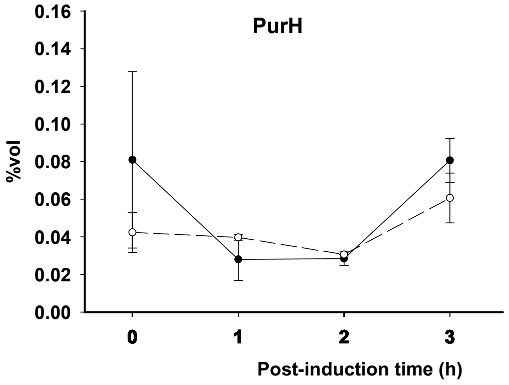

Carbohydrate metabolic and catabolic process (20-23)

20.

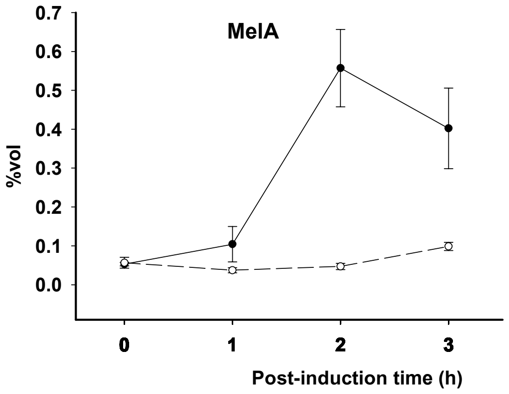

21.

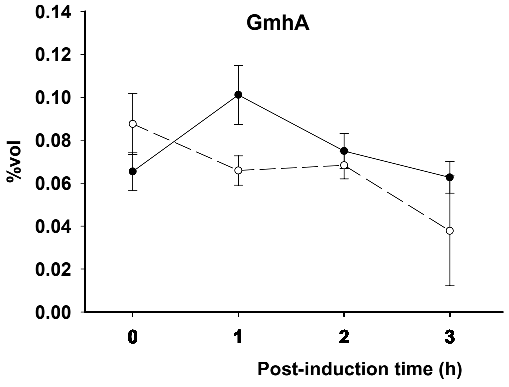

22.

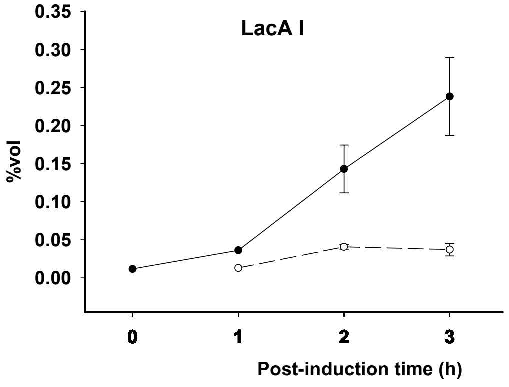

23.

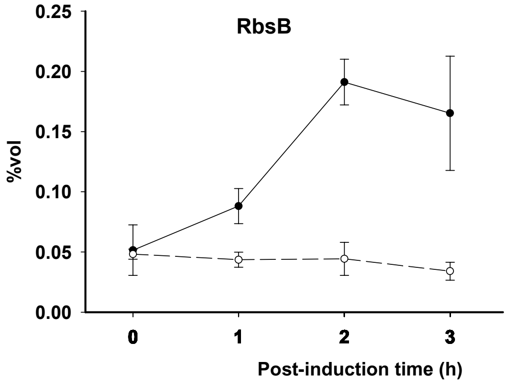

Pentose phosphate pathway (24)

24.

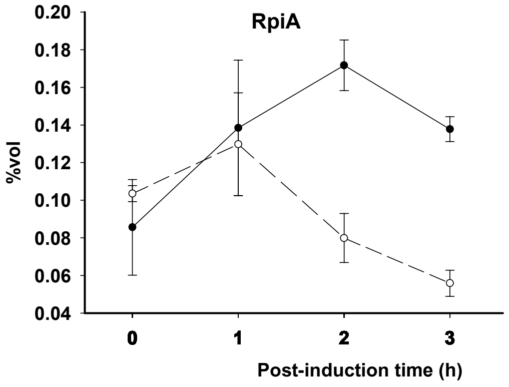

Other function and pathway (26-43)

26.

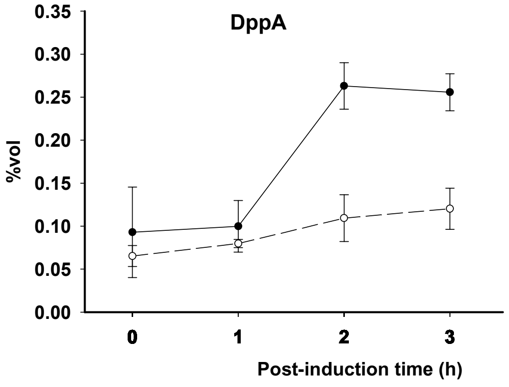

27.

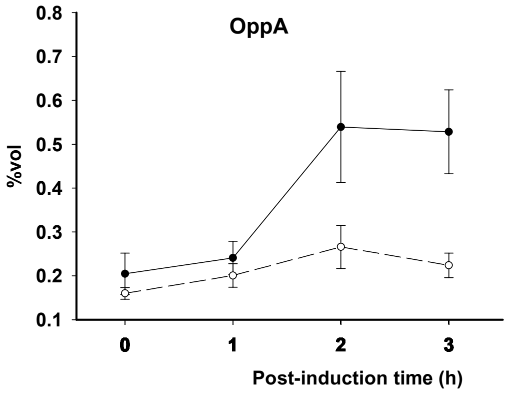

28.

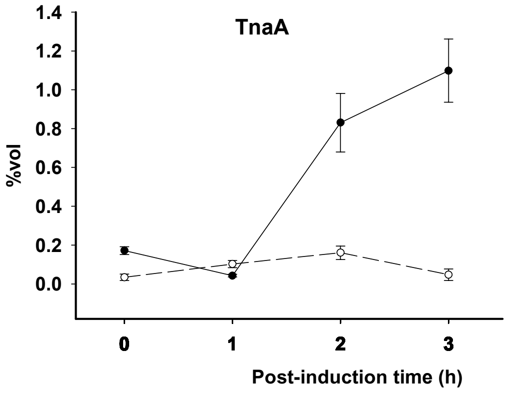

29.

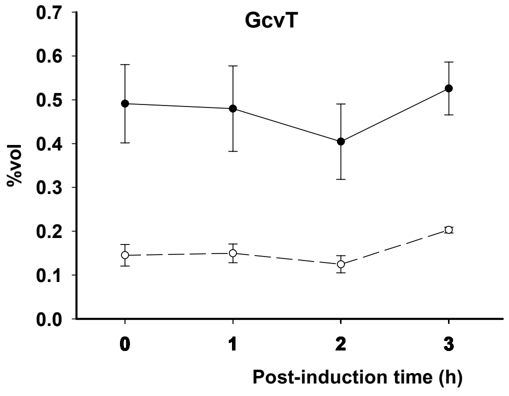

30.

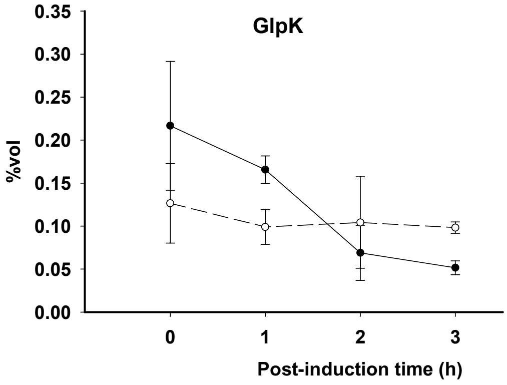

31.

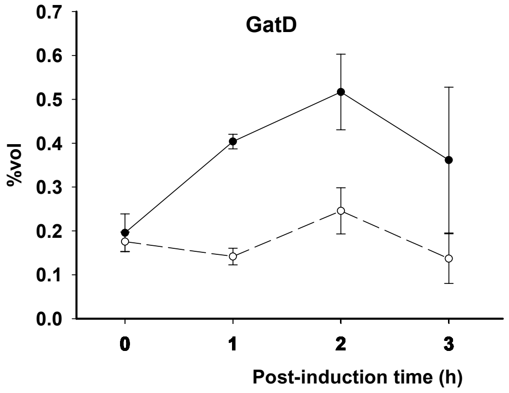

32.

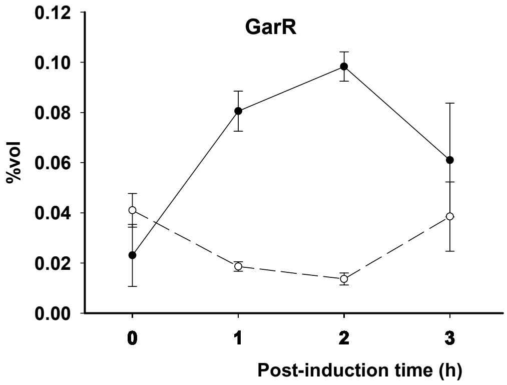

33.

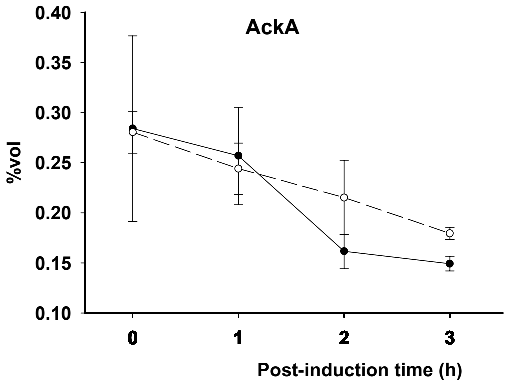

34.

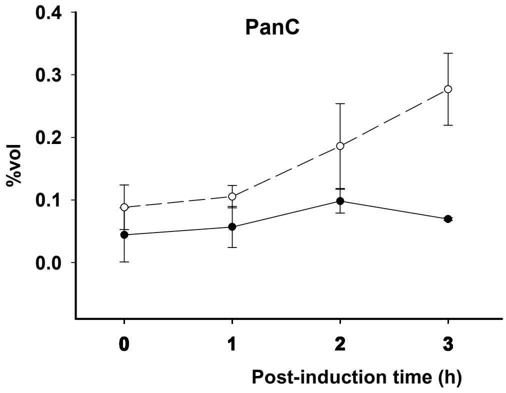

35.

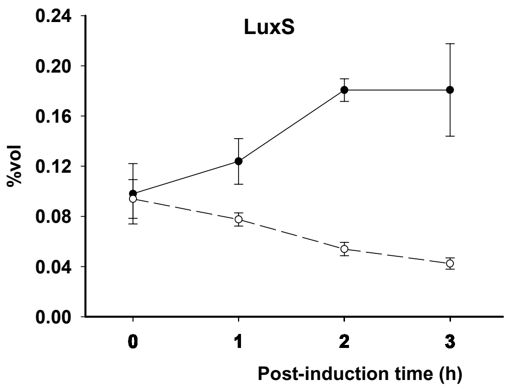

36.

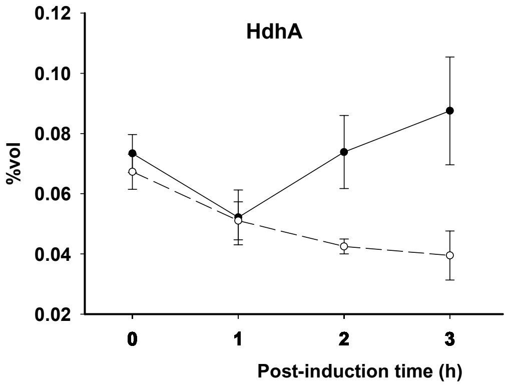

37.

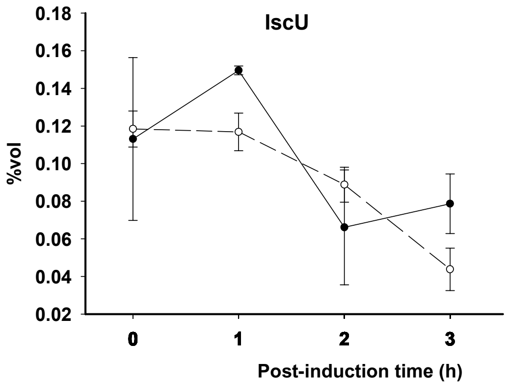

38.

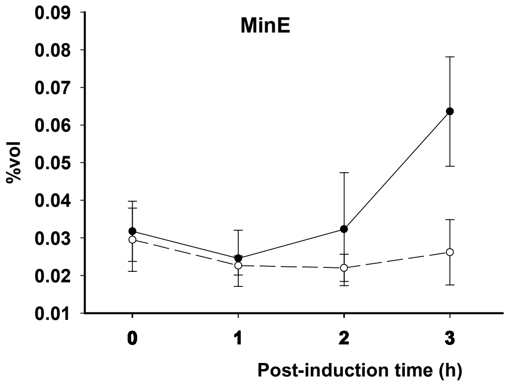

39.

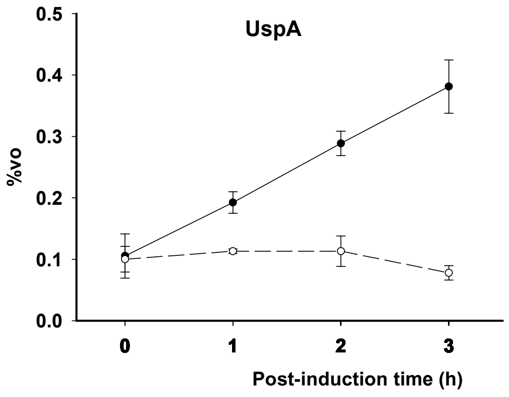

40.

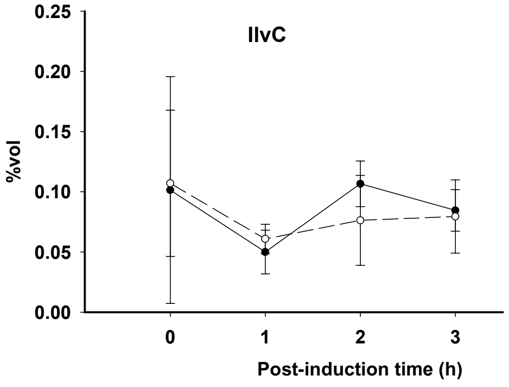

41.

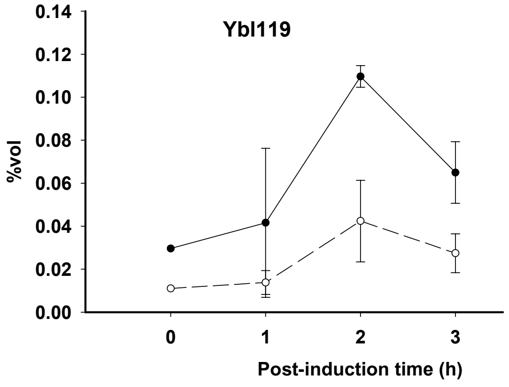

42.

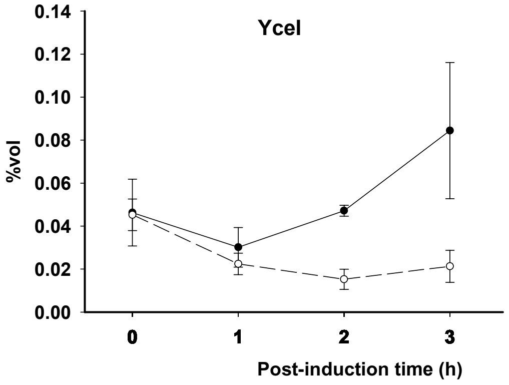

43.

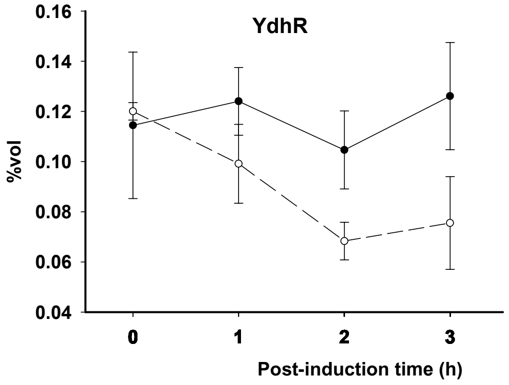

Antibiotics resistance (44-49)

44.

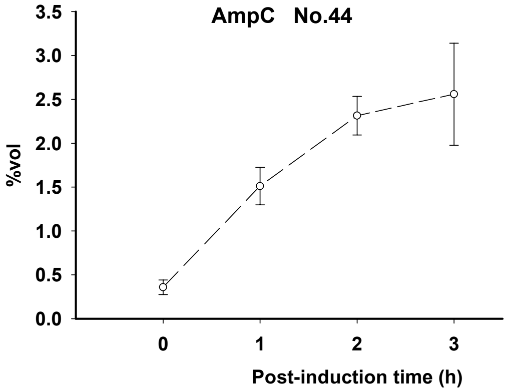

45.

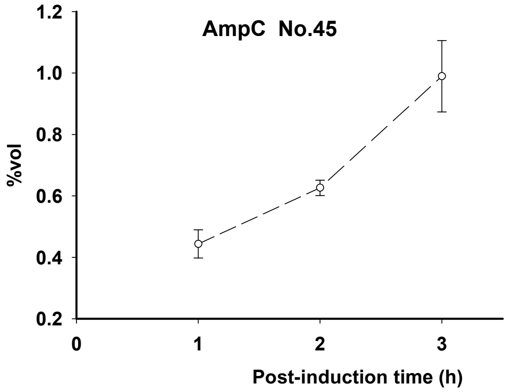

46.

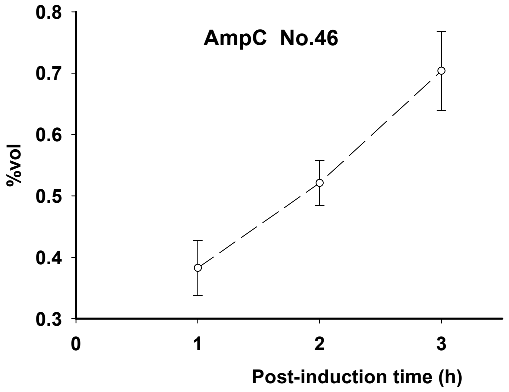

47

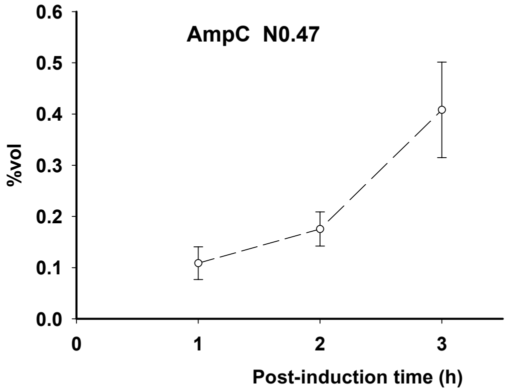

48.

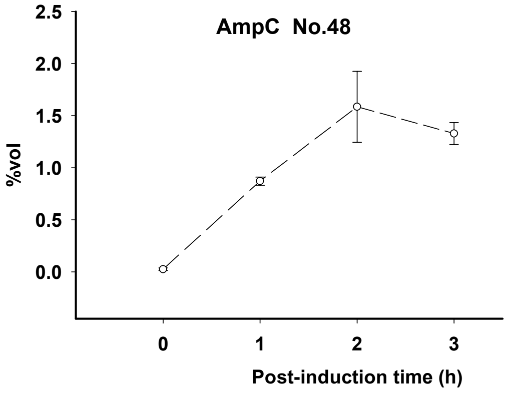
AmpC No.48
%vol
2.5
2.0
1.5
1.0
0.5
0.0
0
1
2
3
Post-induction time (h)

49.

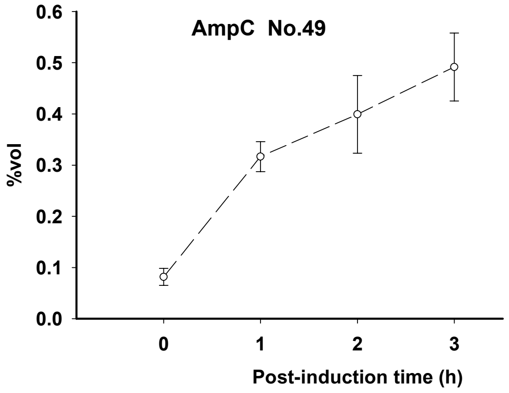
AmpC No.49
%vol
0.6
0.5
0.4
0.3
0.2
0.1
0.0
0
1
2
3
Post-induction time (h)
