# Supplementary material for: Protein solubility and differential proteomic profiling of recombinant Escherichia coli overexpressing double-tagged fusion proteins
Source: Microb Cell Fact. 2010 Aug 28;9:63. doi: 10.1186/1475-2859-9-63 (PMC2940792; doi:10.1186/1475-2859-9-63)

Supplemental Figure 4: Assay of tagged proteins by SDS-PAGE. SDS-PAGE of protein extracts from *E. coli* BL21 overexpressing double-tagged GST-Neu5Ac aldolase-5R (A) and single-tagged GST-Neu5Ac aldolase (B) after 3 h of IPTG induction. Lane M: protein marker; lanes 1-3: the first, second and third extractions from cell pellets; lane P: extraction from aggregates as described in the Methods section. The double-tagged GST-Neu5Ac aldolase-5R was expressed in bacteria harboring pGEX-2TK-nanA-5R; while the single-tagged GST-Neu5Ac aldolase was expressed in bacteria harboring plasmid pGEX-1λT with an inserted sequence coding for Neu5Ac aldolase. Arrows indicate double-tagged GST-Neu5Ac aldolase-5R (in A) and single-tagged GST-Neu5Ac aldolase (in B).

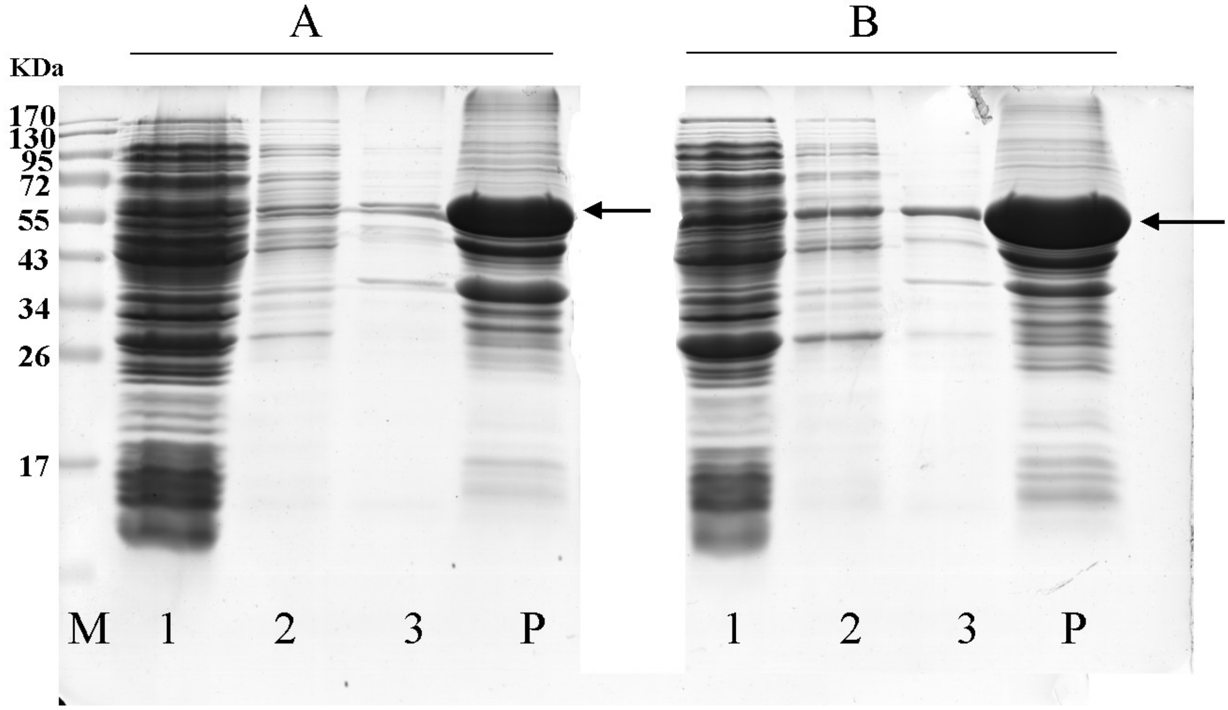

Supplement: Additional file 4 — Supplemental Figure 4: Assay of tagged proteins by SDS-PAGE. SDS-PAGE of protein extracts from E. coli BL21 overexpressing double-tagged GST-Neu5Ac aldolase-5R (A) and single-tagged GST-Neu5Ac aldolase (B) after 3 h of IPTG induction. Lane M: protein marker; lanes 1-3: the first, second and third extractions from cell pellets; lane P: extraction from aggregates as described in the Methods section. The double-tagged GST-Neu5Ac aldolase-5R was expressed in bacteria harboring pGEX-2TK-nanA-5R; while the single-tagged GST-Neu5Ac aldolase was expressed in bacteria harboring plasmid pGEX-1λT with an inserted sequence coding for Neu5Ac aldolase. Arrows indicate double-tagged GST-Neu5Ac aldolase-5R (in A) and single-tagged GST-Neu5Ac aldolase (in B). [file 1475-2859-9-63-S4.PDF]
